# Mitochondria–plasma membrane contact sites regulate the ER–mitochondria encounter structure

**DOI:** 10.1242/jcs.263685

**Published:** 2025-02-18

**Authors:** Jason C. Casler, Clare S. Harper, Laura L. Lackner

**Affiliations:** Department of Molecular Biosciences, Northwestern University, Evanston, IL 60208, USA

**Keywords:** Membrane contact sites, Mitochondria, Mitochondrial positioning

## Abstract

Cells form multiple, molecularly distinct membrane contact sites (MCSs) between organelles. Despite knowing the molecular identity of several of these complexes, little is known about how MCSs are coordinately regulated in space and time to promote organelle function. Here, we examined two well-characterized mitochondria–endoplasmic reticulum (ER) MCSs – the ER–mitochondria encounter structure (ERMES) and the mitochondria–ER–cortex anchor (MECA) in *Saccharomyces cerevisiae*. We report that loss of MECA results in a substantial reduction in the number of ERMES contacts. Rather than reducing ERMES protein levels, loss of MECA results in an increase in the size of ERMES contacts. Using live-cell microscopy, we demonstrate that ERMES contacts display several dynamic behaviors, such as *de novo* formation, fusion and fission, that are altered in the absence of MECA or by changes in growth conditions. Unexpectedly, we find that the mitochondria–plasma membrane (PM) tethering, and not the mitochondria–ER tethering, function of MECA regulates ERMES contacts. Remarkably, synthetic tethering of mitochondria to the PM in the absence of MECA is sufficient to rescue the distribution of ERMES foci. Overall, our work reveals how one MCS can influence the regulation and function of another.

## Teaser

**Summary:** Mitochondria–plasma membrane contact sites regulate the distribution and dynamics of mitochondria–ER membrane contact sites by controlling the positioning of the mitochondrial network.

## INTRODUCTION

The formation of membrane contact sites (MCSs) is a ubiquitous mechanism by which cells organize the interface between two or more organelles to perform specific biological functions (Scorrano et al., 2019). The functions of MCSs are highly diverse, ranging from the nonvesicular exchange of biomolecules, such as lipids, to broader roles in regulating organelle dynamics and positioning (Prinz et al., 2020). Although MCSs have been identified between nearly every organelle pair tested, few are characterized at the molecular level. Moreover, despite evidence that organelles make multiple molecularly and functionally distinct contacts within a cell, little is known about how specific contacts affect the formation or function of others. Thus, characterizing how MCSs influence one another is a crucial next step towards understanding the role of MCSs in maintaining organelle homeostasis. To begin to approach this problem, we aimed to dissect how two well-characterized mitochondrial membrane contact sites, the endoplasmic reticulum (ER)–mitochondria encounter structure (ERMES) and the mitochondria–ER–cortex anchor (MECA), are coordinately regulated.

Owing to its compact genome and well-defined organelle morphology, work in *Saccharomyces cerevisiae* has led the field in identifying the molecular components of MCSs. A landmark study used a synthetic biology screen to identify a mitochondria–ER tethering complex, namely ERMES (Kornmann et al., 2009). ERMES consists of four protein subunits – Mmm1, Mdm34, Mdm10 and Mdm12. Mmm1 contains a transmembrane domain and resides in the ER membrane, Mdm34 and Mdm10 associate with the mitochondrial outer membrane, and Mdm12 is cytosolic (Kornmann et al., 2009). Three subunits, Mmm1, Mdm34 and Mdm12, contain synaptotagmin-like mitochondrial lipid-binding protein (SMP) domains, which are thought to be involved in transporting phosphatidylserine or phosphatidylethanolamine between mitochondria and the ER (Kawano et al., 2018; Wozny et al., 2023). Loss of ERMES results in severe mitochondrial morphology defects and a loss of mitochondrial respiratory capacity (Kornmann et al., 2009; Lang et al., 2015). In addition to the core components of ERMES, several regulators have been identified. Gem1, a GTPase and homolog of the mammalian Miro proteins, physically interacts with the ERMES complex and plays a role in regulating the size and number of ERMES foci (Kornmann et al., 2011). Additional factors, such as Tom7 and Mco6, have been proposed to regulate ERMES, although the precise mechanisms are unclear (Ellenrieder et al., 2016; Rasul et al., 2021). The number of ERMES foci are upregulated during respiratory growth, highlighting its important role in promoting mitochondrial function (Hönscher et al., 2014). In addition, alterations to mitochondrial fusion and fission dynamics as well as the induction of ER stress alter the distribution of ERMES (Kakimoto-Takeda et al., 2022). Finally, defects associated with the loss of ERMES can be suppressed by the expression of gain-of-function alleles of the lipid transport protein Vps13 (Lang et al., 2015). Thus, the wealth of knowledge about the molecular makeup and regulatory mechanisms of ERMES makes it an excellent candidate for a comparative study of MCSs.

Another well characterized MCS is the mitochondria–ER–cortex anchor (MECA). MECA consists of three components – Num1, Mdm36 and Scs2 (Casler et al., 2024; Cerveny et al., 2007; Hammermeister et al., 2010; Klecker et al., 2013; Lackner et al., 2013). Num1 is a large 313 kDa protein that contains two distinct lipid-binding domains – an N-terminal CC domain that binds cardiolipin on the mitochondrial outer membrane (MOM) and a C-terminal PH domain that binds phosphatidylinositol-4,5-bisphosphate [PI(4,5)P_2_] on the plasma membrane (PM). Mdm36 interacts directly with Num1 to promote oligomerization and cortical cluster formation (Hammermeister et al., 2010; Ping et al., 2016; Won et al., 2021). ER tethering is mediated by an interaction between a C-terminal two phenylalanines in an acidic tract (FFAT) motif in Num1 and the integral ER protein Scs2, a homolog of the mammalian VAP proteins (Casler et al., 2024). MECA forms three to five stable clusters on the cell cortex that anchor mitochondria to the PM and ER. Loss of MECA results in a collapse of the mitochondrial network into the center of the cell and defects in the retention of mitochondria in the mother cell (Cerveny et al., 2007; Klecker et al., 2013; Lackner et al., 2013). Coupled to its role in organelle tethering, MECA regulates mitochondrial division, phosphatidylinositol-4-phosphate metabolism and the positioning of the mitotic spindle by anchoring dynein at the cell cortex (Anderson et al., 2022; Casler et al., 2024; Farkasovsky and Küntzel, 1995; Harper et al., 2023; Omer et al., 2018). Thus, MECA is a multifunctional MCS that serves as a crucial organizational hub that integrates multiple cellular signals to influence organelle function. Despite detailed knowledge about the molecular composition and functions of MECA, we know little about how this tripartite MCS influences the function of other MCSs.

In this work, we sought to identify how MECA and ERMES are coordinately regulated. Our results reveal that, unexpectedly, mitochondria–PM tethering plays a role in regulating the number and size of ERMES contacts. Furthermore, controlling the extent of mitochondria–PM tethering might be a general mechanism to regulate the distribution and function of mitochondrial MCSs.

## RESULTS

### Mitochondria–PM tethering by MECA regulates the size and number of ERMES foci

To begin to test whether MECA and ERMES are coordinately regulated, we wanted to first examine how loss of one MCS affects the other. Previous studies have demonstrated that Num1, the core tethering component of MECA, associates with mitochondria in the absence of ERMES (Cerveny et al., 2007). However, the severe alterations in mitochondrial morphology and function as well as negative effects on cellular fitness observed in ERMES mutants make it difficult to disentangle specific effects of the loss of ERMES contacts on MECA function beyond mitochondrial tethering (Burgess et al., 1994; Kornmann et al., 2009). Thus, we decided to test whether loss of MECA influences ERMES function.

First, we generated *Saccharomyces cerevisiae* strains expressing C-terminally tagged ERMES subunits from their endogenous loci in the presence or absence of Num1. To validate the functionality of the fusion proteins, we determined the ability of the cells to grow on glycerol medium (YPEG), a nonfermentable carbon source that forces cells to respire and requires functional mitochondria. All tags tested, with the exception of Mmm1–Halo, had no obvious growth defect on dextrose or glycerol-containing medium (Fig. S1A). Mmm1–Halo displayed a slight but reproducible growth defect on YPEG, indicating that the tag might partially compromise Mmm1 function (Fig. S1A). Next, we compared the localization of tagged ERMES components using fluorescence microscopy. As expected, all tagged versions of Mdm34, Mdm12 and Mmm1 displayed excellent colocalization and a similar number of foci per cell (Fig. S1B,C). Unexpectedly, however, in ∼25% of cells expressing tagged versions of Mmm1, we observed Mmm1 foci that did not colocalize with other ERMES components nor associate with mitochondria (Fig. S1D,E). These foci might be nonfunctional aggregates and/or indicative of a stress response. Alternatively, Mmm1 could have additional functions distinct from its role in the ERMES complex. Based on these results, we conclude that C-terminally tagged versions of Mdm12 and Mdm34 are suitable ERMES reporters.

**Fig. 1.**
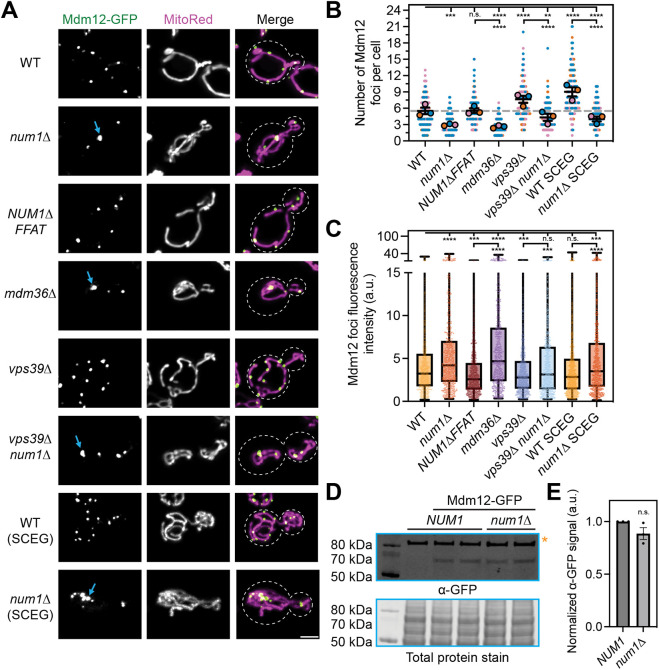
**Loss of MECA reduces the number of ERMES foci.** (A) Super-resolution fluorescence micrographs of cells expressing Mdm12–GFP and the mitochondrial matrix marker MitoRed in the indicated genetic backgrounds. Cells were grown in SCD (minimal dextrose) medium, except for the bottom two panels which were grown in SCEG (minimal ethanol glycerol) media. Individual channels are shown in grayscale. Blue arrows indicate large Mdm12 foci. Cell outlines are indicated by white dashed lines. Scale bar: 2 µm. (B) Quantification of the number of Mdm12 foci per cell from strains shown in A. A detailed description of how this quantification was performed can be found in the Materials and Methods section. Each dot represents an individual cell, and replicates are represented by different colors. The mean of each replicate is shown as an outlined dot of the appropriate color. Error bars indicate s.e.m. of the three replicate averages. Each replicate contained at least 45 cells for a total of 125–215 cells per condition. A dashed gray line indicates the WT average to aid visual comparison. n.s., not significant; ****P<0.0001; ***P<0.001; **P<0.01 (ordinary one-way ANOVA with multiple comparisons). (C) Quantification of the Mdm12 foci fluorescence intensity from the strains shown in A. Each dot represents the fluorescence intensity of an individual Mdm12 foci. The whiskers on the box-and-whisker plots extend to the minimum and maximum values; the box extends from the 25th–75th percentiles with the median indicated. ****P<0.0001; ***P<0.001 (ordinary one-way ANOVA with multiple comparisons). (D) Western blot analysis of steady state Mdm12 protein levels in wild-type and *num1*Δ cells. A negative control and two technical replicates of cells expressing Mdm12–GFP in wild-type and *num1*Δ backgrounds are shown. The orange asterisk indicates a nonspecific band. The blot is representative of three biological replicates. (E) Quantification of the western blot shown in D and two additional replicates. For each replicate, the intensity of the Mdm12–GFP band was quantified and normalized to the signal from the total protein stain. The normalized Mdm12–GFP signal from technical replicates was averaged and normalized to the signal from WT cells for comparison. Each dot represents one replicate. Error bars are s.e.m. n.s., not significant (unpaired two-tailed *t*-test).

To determine the effect of loss of MECA on ERMES function, we examined the number and size of ERMES foci in MECA mutants. A previous study performed a genetic screen using changes in the number of ERMES foci to identify ERMES regulators (Elbaz-Alon et al., 2014). Interestingly, in that screen, Num1 was identified as a protein whose loss affected the appearance of ERMES foci. In a wild-type background under fermentative growth conditions, cells displayed an average of ∼5.5 Mdm12 foci per cell, and these were similar in size and distributed throughout the mitochondrial network (Fig. 1A,B). Remarkably, loss of Num1, the core tethering component of MECA, resulted in a substantial decrease in the number of Mdm12 foci per cell (from ∼5.5 to ∼2.9) (Fig. 1A,B). Importantly, loss of Num1 did not completely destabilize the ERMES complex, as both Mdm34 and Mmm1 remained colocalized with Mdm12 in *num1*Δ cells (Fig. S1B,C). Beyond a reduction in the total number of ERMES foci in *num1*Δ cells, we also observed ERMES foci that were larger than foci in wild-type cells (Fig. 1A, blue arrow). Quantification of this phenotype revealed that loss of Num1 results in a significant increase in the fluorescence intensity of ERMES foci (Fig. 1C). To determine whether the reduced number of ERMES foci was due to changes in protein levels, we performed western blotting to compare the amount of Mdm12 in wild-type and *num1*Δ cells. Mdm12 protein levels in Num1 mutants were similar to those in wild type (Fig. 1D,E). Thus, the reduction in the number of ERMES foci might result from a change in distribution rather than a loss of ERMES tethers.

We next wanted to determine whether the mitochondrial and ER-tethering functions of Num1 are required to maintain the distribution of ERMES foci. Previously, we have demonstrated that loss of the MECA component Mdm36 disrupts Num1 oligomerization and significantly reduces the number of mitochondria–PM tethering points (Lackner et al., 2013; Ping et al., 2016). More recently, we have demonstrated that deletion of the Num1 FFAT motif disrupts Num1 ER association without compromising its ability to function as a mitochondria–PM tether (Casler et al., 2024). Therefore, we examined ERMES foci in cells expressing Num1ΔFFAT or in cells lacking Mdm36. Unexpectedly, cells expressing Num1ΔFFAT showed no difference in the number of Mdm12 foci whereas those lacking Mdm36 phenocopied a *NUM1* deletion (Fig. 1A,B). Thus, Num1 likely regulates the number ERMES foci by controlling the cortical distribution of the mitochondrial network rather than through its function as a mitochondria–ER tether. Importantly, the Num1ΔFFAT experiment also suggests that the regulation of ERMES foci number by Num1 is unrelated to the well-characterized role of Num1 in promoting mitochondrial division (Cerveny et al., 2007; Klecker et al., 2013). Recently, we have demonstrated that, despite maintaining cortically tethered mitochondrial networks, cells expressing Num1ΔFFAT exhibit a reduced rate of mitochondrial division that is similar to that in cells lacking Num1 (Casler et al., 2024). Therefore, Num1ΔFFAT operates as a separation-of-function allele that cleanly disentangles the functions of Num1 in mitochondrial division and mitochondria–PM tethering as well as the functions of Num1 in mitochondrial division and regulation of ERMES, as demonstrated here. Although the complete loss of the mitochondrial division machinery has been reported to alter the distribution of ERMES foci, our results suggest that mitochondria–PM tethering constitutes a novel mechanism that regulates ERMES in a manner that is unrelated to alterations in mitochondrial dynamics (Kakimoto-Takeda et al., 2022).

Previously, we have demonstrated that misregulated dynein contributes to the respiratory growth defect seen in cells lacking Num1 (White et al., 2022). Therefore, we wanted to rule out the possibility that the reduction in the number of ERMES foci was due to misregulation of dynein anchoring in *num1*Δ cells. Deleting *DYN1* from wild-type and *num1*Δ cells caused no additional change to the number of Mdm12 foci, making it unlikely that the role of Num1 as a dynein anchor significantly affects ERMES regulation (Fig. S2A,B). Together, our data support the conclusion that it is the function of Num1 in mitochondria–PM tethering that is distinctly required to maintain the wild-type number of ERMES foci.

To expand on these results, we also examined the distribution of ERMES foci in cells lacking Num1 in combination with conditions that are known to upregulate ERMES. Consistent with previous reports, we found that the loss of the homotypic fusion and protein sorting (HOPS) component Vps39 and growth on glycerol, a nonfermentable carbon source, significantly increased the number of ERMES foci (Elbaz-Alon et al., 2014; Hönscher et al., 2014). Remarkably, loss of MECA reduced the number of ERMES foci by nearly half in both conditions (Fig. 1A,B). Thus, MECA likely regulates the number of ERMES foci via a mechanism distinct from that of Vps39 or growth in glycerol.

To determine whether this phenotype was only related to the core ERMES machinery, we also examined the localization of Lam6, a lipid transport protein that is known to associate with ERMES and other MCSs, in Num1 mutants (Elbaz-Alon et al., 2015; Murley et al., 2015). Consistent with previous reports, Lam6 displayed ∼6.5 foci per cell, with most being mitochondria associated (Fig. S2C,D). Interestingly, loss of Num1 resulted in a similar reduction in the number of Lam6 foci to that seen for Mdm12 (Fig. S2C,D).

### ERMES contacts move with the mitochondrial network and display dynamic behaviors like fusion, fission and *de novo* formation

**Fig. 2.**
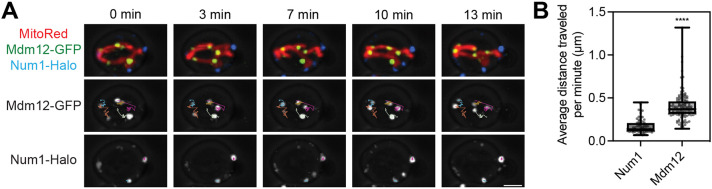
**ERMES foci move with the mitochondrial network.** (A) Fluorescence micrographs from Movie 1 of cells expressing MitoRed, Mdm12–GFP and Num1–Halo. Individual channels are shown in grayscale. Images are overlayed with a brightfield image to indicate the cell boundaries. Individual Mdm12 and Num1 foci were tracked throughout the course of the video using the TrackMate plugin in Fiji. The movement of individual foci is depicted as a colored line overlayed on the grayscale images. Scale bars: 2 µm. (B) Quantification of the average distance traveled of individual Num1 or Mdm12 foci per minute. The whiskers on the box-and-whisker plots extend to the minimum and maximum values; the box extends from the 25th–75th percentiles with the median indicated. ****P<0.0001 (unpaired two-tailed *t*-test).

Owing to the fact that the number of ERMES foci was reduced but protein levels remained constant, we reasoned that loss of Num1 might cause a redistribution of existing ERMES contacts. Previously, we and others have demonstrated that MECA contacts are stable structures that display little intracellular movement or dynamic exchange with unassembled subunit pools (Amado et al., 2023; Kraft and Lackner, 2017). New MECA foci form in the growing bud whereas foci that are already assembled remain in the mother (Kraft and Lackner, 2017). Although FRAP studies indicate that ERMES foci are also stable structures, there has yet to be a detailed analysis of how new ERMES foci are formed and maintained (Amado et al., 2023; Nguyen et al., 2012). Therefore, we used 4D confocal microscopy to image cells expressing Mdm12–GFP, Num1–Halo and the mitochondrial matrix marker MitoRed to compare the dynamics of ERMES contacts to those of MECA. Although both contact site proteins remained associated with the mitochondrial network throughout the movies, ERMES foci were strikingly far more mobile than MECA foci (Fig. 2A,B; Movie 1). We used the TrackMate plugin for the image processing package Fiji to segment and track the movement of individual Mdm12 and Num1 foci throughout the course of 15-min movies (Ershov et al., 2022). While Num1 foci were mostly stationary, Mdm12 foci moved an average of ∼0.4 µm per minute. Thus, in contrast to the stationary mitochondria–PM anchor points generated by MECA, ERMES contacts move with the mitochondrial network.

**Fig. 3.**
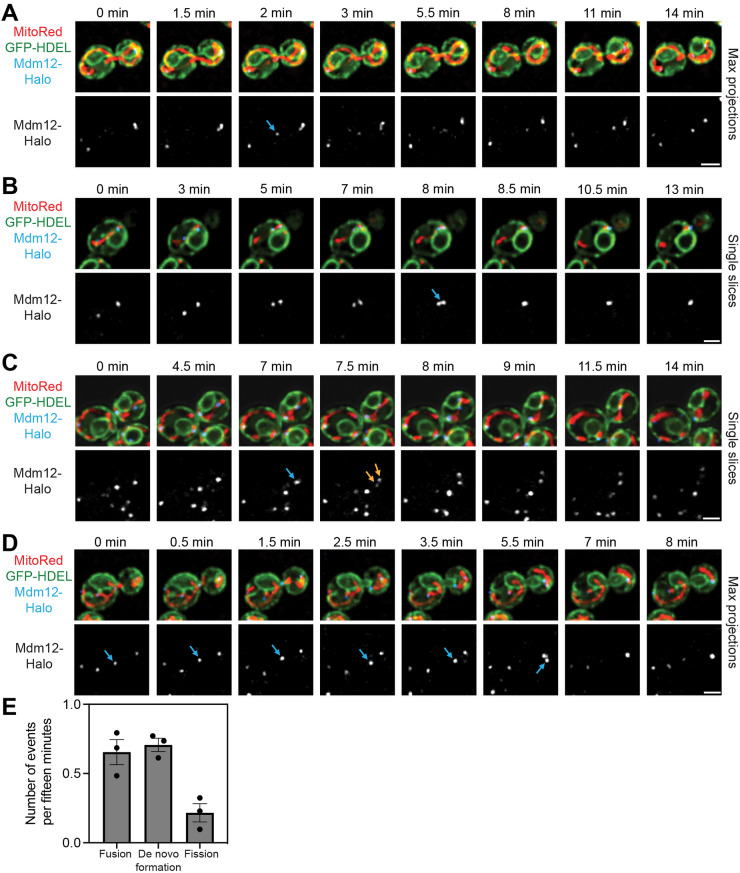
**ERMES foci display dynamic behaviors.** Cells expressing Mdm12–Halo, the general ER marker GFP–HDEL and MitoRed were grown to mid-log phase and imaged via 4D confocal microscopy. A–D are still frames from Movies 2–5 representing dynamic behaviors of Mdm12. The Mdm12–Halo channel is shown in grayscale. (A) The blue arrow points to the *de novo* formation of a new Mdm12–Halo focus that persists throughout the rest of the movie. Images are max projections of a full Z-stack. (B) An example of two distinct ERMES foci that fuse and remain associated. The blue arrow indicates the frame when the foci first come in contact. Images are single slices from a Z-stack. (C) An example of a single Mdm12 focus (blue arrow) splitting into two smaller foci (orange arrows) that remain separated. Images are single slices from a Z-stack. (D) Blue arrows indicate an example of an Mdm12 focus being transported from the mother to the daughter cell. Images are max projections of a full *Z*-stack. Scale bars: 2 µm. (E) Quantification of the frequency of observed Mdm12 fusion, *de novo* formation and fission events. A detailed description of the quantification methodology can be found in the Materials and Methods section. Each dot represents one replicate (*n*=3 imaging replicates; 33–34 cells per replicate for 100 total cells). Error bars are mean±s.e.m.

Next, we wanted to analyze how the number of ERMES foci is maintained as cells grow and divide. To do so, we used 4D confocal microscopy to image cells expressing the mitochondrial matrix marker MitoRed, the general ER marker GFP–HDEL and Mdm12–Halo. To our surprise, Mdm12 foci exhibited a series of dynamic behaviors. First, we were able to track the apparent *de novo* formation of new ERMES foci at sites that contained both mitochondrial and ER membranes (Fig. 3A; Movie 2). A cryo-EM study has previously reported that typical ERMES foci seen by confocal microscopy contain ∼24 tethers (Wozny et al., 2023). Therefore, the *de novo* formation events visualized in our movies might represent the coalescence of individual or small groups of tethers into a larger cluster that forms a stable contact site. In addition to *de novo* formation events, we also witnessed apparent fusion and fission events (Fig. 3B,C; Movies 3,4). For the former, distinct Mdm12-positive foci would approach, fuse and remain associated over extended periods of time. For the latter, we observed what appeared to be individual Mdm12 foci break apart into two distinct smaller foci that remained separated. Occasionally, ERMES foci would appear to slide along the surface of the mitochondrial membrane (Fig. 3B; Movie 3). Without a fiduciary marker, however, it is impossible to confidently state that the MCS is moving independently of the underlying mitochondrial membrane. Finally, we also documented the movement of Mdm12 foci from the mother cell to the daughter cell (Fig. 3D; Movie 5). In a particularly striking example, we observed the inheritance of an ERMES focus from the mother cell coupled to nuclear and mitochondrial inheritance (Fig. 3D; Movie 5). Quantification of these events revealed that ∼0.65 fusion events, ∼0.71 *de novo* formation events and ∼0.22 fission events can be detected in the 15-min timeframe of our movies (Fig. 3E). To ensure that our analysis of Mdm12 was applicable to the entire ERMES complex, we also analyzed the dynamics of Mdm34 foci. We were able to capture the *de novo* formation, fusion and fission, and inheritance of Mdm34 foci at similar frequencies to Mdm12 (Fig. S3A–E; Movies 6–9). Importantly, our quantification of these dynamic events is limited by the threshold of detection of our imaging system. It is likely our analysis failed to capture some *de novo* formation events due to photobleaching during the course of the movie. Similarly, fission events where only a minor amount of ERMES tethers were separated from a larger focus might also have been missed. Notably, we never detected the complete disassembly or degradation of a preexisting ERMES focus using either Mdm12 or Mdm34 as a marker. Thus, the total number of ERMES foci per cell is likely determined by a combination of *de novo* formation, fusion, fission and inheritance events. Although the frequency of these dynamic events is fairly low, the result is a net increase in the number of ERMES foci as the cell cycle progresses.

### ERMES dynamics are altered by growth conditions and loss of MECA

**Fig. 4.**
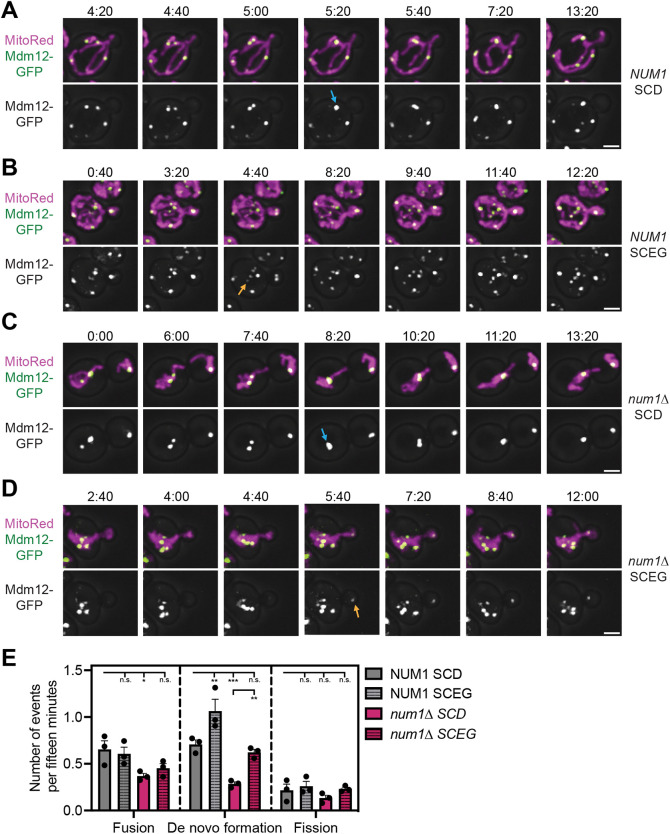
**Loss of MECA and changes in growth conditions regulate ERMES foci dynamics.** (A–D) Images from Movie 10 depicting Mdm12–GFP and MitoRed in *NUM1* or *num1*Δ cells grown in SCD or SCEG. Images are maximum projections of a full *Z*-stack. The Mdm12–GFP channel is shown in grayscale. Images are overlayed with a brightfield image to indicate the cell boundaries. Blue arrows highlight ERMES foci fusion events and orange arrows highlight *de novo* formation events. Scale bars: 2 µm. (E) Quantification of the frequency of observed Mdm12 dynamic events in *NUM1* or *num1*Δ cells grown in SCD or SCEG. ***P<0.001; **P<0.01; *P<0.05; n.s., not significant (ordinary one-way ANOVA with multiple comparisons). Each dot represents one replicate (*n*=3 imaging replicates; 33–34 cells per replicate for 100 cells total). Error bars are mean±s.e.m. Data from wild-type cells in SCD is the same as Fig. 3E and is included to aid visual comparison.

Next, we probed how ERMES dynamics are affected by growth conditions and loss of MECA. We expressed Mdm12–GFP with MitoRed in wild-type and *num1*Δ cells and imaged them via 4D confocal microscopy in dextrose- or glycerol-containing medium. In all conditions, we observed fusion, fission and the *de novo* formation of ERMES foci (Fig. 4A–E; Movie 10). Interestingly, growth in glycerol increased the rate of *de novo* formation of ERMES foci by ∼2-fold in both wild-type and *num1*Δ cells while fusion and fission rates remained similar (Fig. 4B,D,E). Loss of MECA resulted in a nearly two-fold reduction of all dynamic events (Fig. 4C–E). The differences in the rate of *de novo* formation events are consistent with the differences in the steady state number of ERMES foci observed in each condition. A ∼2-fold increase in the *de novo* formation rate of ERMES foci in wild-type cells grown in glycerol corresponds to a nearly two-fold increase in the number of ERMES foci (∼5.5 to ∼9.0). Similarly, loss of Num1 results in an approximate two-fold decrease in the *de novo* formation rate and an approximate two-fold decrease in the number of ERMES foci (∼5.5 to ∼2.9). Thus, the change in the steady state number of ERMES foci in different conditions can be explained by changes in the rate of formation of new foci.

### Reformation of MECA restores the distribution of ERMES foci over several hours

**Fig. 5.**
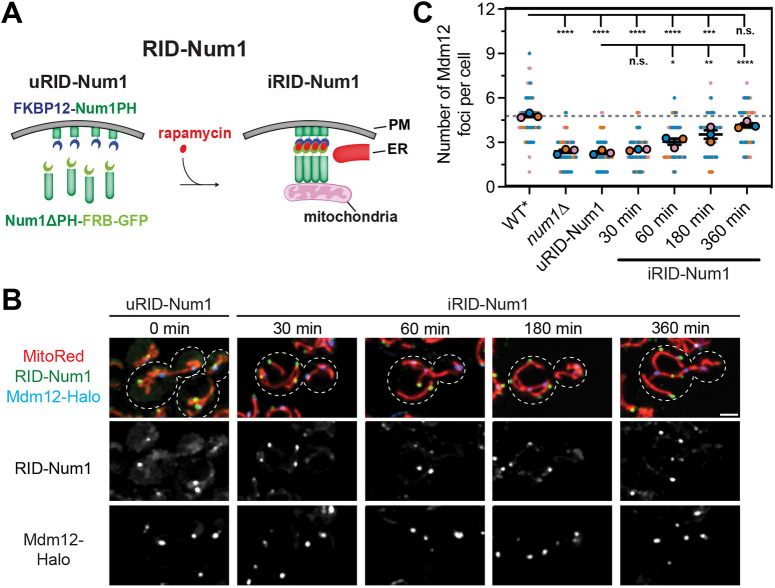
**Induced formation of MECA contacts restores the number of ERMES foci over several hours.** (A) Cartoon depiction of the RID-Num1 system. Num1ΔPH-FRB–GFP is expressed from the endogenous locus and FKBP12–Num1PH is expressed from an integrated plasmid. Upon addition of rapamycin, FKBP and FRB dimerize and the full Num1 molecule is reconstituted. Num1 then oligomerizes and forms stable mitochondria–ER–PM tethering points (Harper et al., 2023). (B) Fluorescence micrographs of RID-Num1 cells expressing MitoRed and Mdm12–Halo before and up to 360 min after the introduction of rapamycin. The RID-Num1 and Mdm12–Halo channels are shown in grayscale. Cell outlines are indicated by the white dashed lines. Images are full projections of a *Z*-stack. Scale bar: 2 µm. (C) Quantification of the number of Mdm12–Halo foci before and after the introduction of rapamycin in RID-Num1 cells. The wild-type strain in this figure is a W303 strain harboring the *tor1-1* and *fpr1*Δ mutations that confer resistance to rapamycin and is denoted as WT*. Quantification is depicted identically to Fig. 1B (*n*=3 imaging replicates, 33–34 cells per replicate for 100 cells total). n.s., not significant; ****P<0.0001; ***P<0.001; **P<0.01; *P<0.05 (ordinary one-way ANOVA with multiple comparisons).

Little is known about how the kinetics of MCS formation results in intracellular changes. To better understand how the formation of MECA influences mitochondrial biology, we previously developed a system to induce the formation of MECA upon addition of a stimulus (Harper et al., 2023). This system, termed RID-Num1 for rapamycin-inducible dimerization of Num1, splits the Num1 tether in half, expressing Num1ΔPH-FRB–GFP from the endogenous *NUM1* locus and an exogenous copy of the Num1 PH domain fused to an FKBP12 tag from an additional locus (Fig. 5A). These experiments are performed in a rapamycin-resistant *tor1-1 fpr1*Δ background (Haruki et al., 2008). Addition of rapamycin reconstitutes the full Num1 molecule via dimerization of Num1ΔPH-FRB–GFP and FKBP12–Num1PH. Reconstituted Num1 molecules then self-associate and form MECA contact sites.

We used the RID-Num1 system to determine how rapidly the distribution of ERMES foci changed upon restoration of MECA. As expected, prior to the addition of rapamycin, the mitochondrial network was collapsed into the center of the cell and on average 2–3 Mdm12 foci were visible (Fig. 5B,C). Consistent with our previous results, within 30 min of rapamycin addition, cortical Num1 foci that tether mitochondria were clearly visible. Interestingly, the number of Mdm12 foci per cell slowly increased between 60 and 360 min after rapamycin addition (Fig. 5B,C). By 360 min, the average number of Mdm12 foci per cell remained slightly lower than in wild type, but the difference was no longer statistically significant. Thus, inducing cortical tethering of the mitochondrial network does not rapidly redistribute existing ERMES contacts, but rather changes the total number of distinct ERMES contacts over a period of time that involves at least one cell doubling. One possibility is that the enlarged ERMES foci in MECA mutants are hyperstable or difficult to remodel. Therefore, rather than breaking apart existing ERMES contacts, the rescue in the total number of ERMES foci might be largely due to the *de novo* formation of new foci. Given that the rates of new contact site synthesis are fairly low even when mitochondria are cortically tethered, it is perhaps not surprising that the rescue takes hours rather than minutes (Fig. 4).

### Synthetic tethering of mitochondria to the PM rescues the distribution of ERMES foci in the absence of MECA

**Fig. 6.**
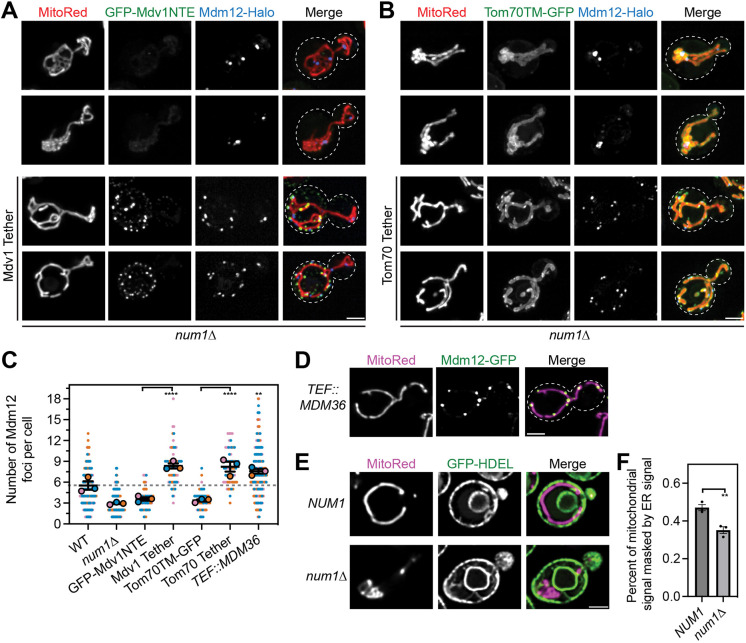
**Synthetic mitochondria-PM tethering increases the number of ERMES foci in the absence of MECA.** (A) Super resolution fluorescence micrographs of cells expressing Mdm12–Halo and MitoRed with GFP–Mdv1NTE without (top images) or with (bottom images) a GFP-nanobody-tagged eisosome component. Two examples of each condition are shown. Individual channels are shown in grayscale. White dashed lines indicate cell outlines. (B) Identical to A except cells expressed Tom70TM–GFP. Scale bars: 2 µm. (C) Quantification of the number of Mdm12 foci in the indicated genetic backgrounds. The WT and *num1*Δ data are duplicated from Fig. 1B to aid visual comparison. Quantification is depicted identically to Fig. 1B (*n*=3 imaging replicates, 33–34 cells per replicate for 100 cells total). ****P<0.0001; **P<0.01 (ordinary one-way ANOVA with multiple comparisons). (D) Fluorescence micrographs of cells expressing MitoRed and Mdm12–GFP in a Mdm36 overexpression background. Mdm36 overexpression was driven by replacing the native promoter with the *TEF* promoter. Quantification is included in C. (E) Super resolution fluorescence micrographs of cells expressing MitoRed and GFP–HDEL in *NUM1* and *num1*Δ backgrounds. Scale bar: 2 µm. Images are single slices from the center of a cell. (F) Quantification of the amount of mitochondrial signal masked by ER signal from cells in E. Each dot represents the average percentage of mitochondrial signal that was masked by ER signal from a single imaging replicate containing between 141 and 248 cells. Error bars represent the mean±s.e.m. of the three imaging replicates. **P<0.01 (unpaired two-tailed *t*-test).

Finally, we wanted to test whether cortical tethering of mitochondria to the PM is sufficient to regulate the number and distribution of ERMES foci. In a previous study, we developed two synthetic systems that tether mitochondria to the PM in the absence of MECA. Both systems target mitochondria to the PM by expressing an anti-GFP nanobody fused to an eisosome component in combination with either a GFP-tagged version of the mitochondrial binding domain of Mdv1 (Mdv1NTE), a protein involved in mitochondrial division, or the transmembrane domain of Tom70 (Tom70TM), a subunit of the mitochondrial import machinery (White et al., 2022). Eisosomes are discrete stable compartments on the PM with poorly characterized functions (Walther et al., 2006). As expected, *num1*Δ cells expressing either GFP–Mdv1NTE or Tom70TM–GFP in the absence of an anti-GFP nanobody-fused eisosome component had collapsed mitochondrial networks and fewer Mdm12 foci than wild-type cells (Fig. 6A–C). Remarkably, expression of either of the synthetic mitochondria–PM tethering systems significantly increased the number of Mdm12 foci (Fig. 6A–C). Unexpectedly, however, the number of Mdm12 foci was higher with the synthetic tethering systems (∼8.2 and ∼8.3 per cell) than in wild-type cells (∼5.5 per cell) (Fig. 6A–C). One potential explanation for this phenomenon could lie in the fact that the synthetic tethering systems likely tether mitochondria to the PM to a higher degree than MECA (White et al., 2022). To test this hypothesis, we measured the number of ERMES foci when Mdm36 was overexpressed – a condition that increases the size and stability of MECA contacts (Casler et al., 2024; Omer et al., 2021). Remarkably, when MECA contacts are enhanced by overexpressing Mdm36, the average number of ERMES foci increased (Fig. 6C,D).

**Fig. 7.**
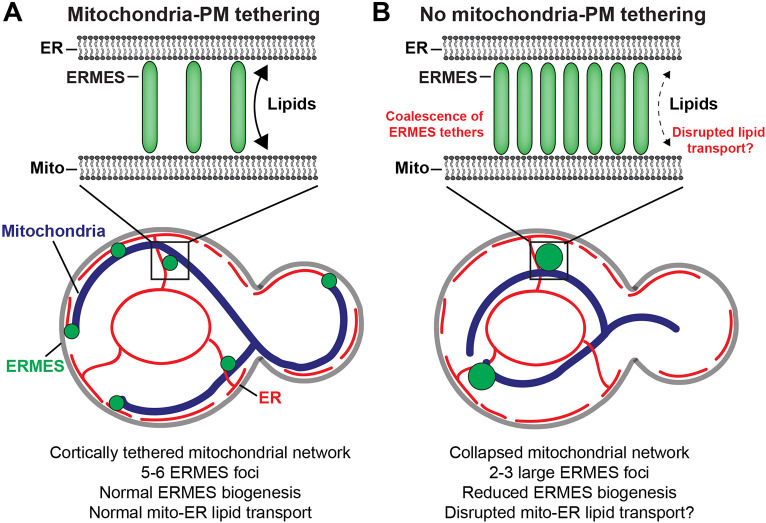
**Mitochondria–PM tethering regulates the distribution and function of ERMES.** (A) In wild-type cells, MECA anchors mitochondria to the cell cortex, which regulates the distribution of ERMES contacts. This scenario might represent the optimal distribution of ERMES contacts to facilitate lipid transport between mitochondria and the ER. (B) In the absence of MECA, the mitochondrial network collapses into the center of the cell and ERMES contacts coalesce into abnormally large structures with an increased number of tethering complexes per foci. This scenario might alter ERMES function, potentially disrupting or decreasing the efficiency of lipid transport between mitochondria and the ER.

Based on these results, we reasoned that mitochondria–PM tethering might influence the total amount of contact between mitochondria and the ER to promote the formation of new MCSs. To test this possibility, we quantified the amount mitochondrial signal that was masked by ER signal in wild-type and *num1*Δ cells. The results demonstrate that loss of mitochondria–PM tethering significantly reduced the fraction of mitochondria in contact with the ER (Fig. 6E,F). Overall, our results support a model in which the primary role of MECA in regulating ERMES is controlling the cortical distribution of the mitochondrial network (Fig. 7).

## DISCUSSION

In this work, we set out to identify how two mitochondrial MCSs are coordinately regulated. We found that loss of MECA results in a substantial decrease in the number of ERMES contacts. Unexpectedly, this was solely related to the mitochondria–PM tethering function of Num1, as mutations that compromise the ability of Num1 to interact with the ER had no effect on the number of ERMES foci (Fig. 1A,B). Remarkably, artificial tethering of the mitochondrial network to the PM was sufficient to rescue ERMES distribution defects in the absence of Num1, strongly implying that Num1 regulates ERMES via its function as a mitochondria–PM tether (Fig. 6A–C). Thus, our work has uncovered a novel function of mitochondria–PM contacts – regulating the distribution and dynamics of mitochondrial MCSs.

How does mitochondria–PM tethering regulate ERMES? In order for a MCS to form, proximity between the organelles is required for tethering to occur. A well-distributed mitochondrial network will have more opportunities to form functional MCSs by increasing the chance it encounters other organelles. This is particularly likely for mitochondria–ER contact sites, such as ERMES, as 20–45% of the PM is occupied by ER in budding yeast (West et al., 2011). Thus, the amount of mitochondria–PM tethering might spatially regulate the formation of mitochondria–ER MCSs. In support of this, conditions that enhance mitochondria–PM tethering, such as growth in nonfermentable carbon sources, overexpression of Mdm36 or expression of synthetic mitochondria–PM tethering systems, increases the number of ERMES foci (Figs 1A–C, 6). In contrast, conditions that reduce mitochondria–PM tethering, such as loss of Num1 or Mdm36, decreases the number of ERMES foci (Figs 1A–C, 7). Furthermore, loss of mitochondria–PM tethering reduces the total amount of mitochondria–ER contact (Fig. 6E,F).

Along with its role in promoting mitochondria–ER MCS formation, cortical mitochondrial tethering is also likely to be important for maintaining the spatial segregation of individual MCSs. A recent, elegant cryo-correlative light and electron microscopy (CLEM) study has revealed that the ERMES foci visualized by confocal microscopy contain ∼20–25 bridge-like structures (Wozny et al., 2023). Despite variability in the spatial arrangement of the ERMES bridges within a contact site, the number of bridges per MCS is fairly consistent. Interestingly, our work has revealed that when the total number of ERMES foci increases during respiratory growth, the intensity of the foci remains consistent (Fig. 1A–C). Taken together, these data suggest that the number of tethers within an ERMES cluster is tightly regulated. Loss of mitochondria–PM tethering disrupts this regulation, resulting in a large number of ERMES tethers coalescing at single location (Fig. 7). Thus, one role of mitochondria–PM tethering might be to distribute the mitochondrial network in a way that prevents the overaccumulation of tethers at one site of contact between two organelles. In the absence of a regulatory mechanism, membrane-tethering complexes will likely be distributed in the most energetically favorable fashion. This could be problematic, as too many or too few tethers could result in hyperstable or unstable contacts with disrupted functions. In support of this, re-establishing mitochondria–PM tethering using the RID-Num1 system only gradually restored the number of ERMES foci (Fig. 5). The enlarged ERMES contacts seen upon loss of MECA might be difficult to remodel and could be less functional than their smaller counterparts (Fig. 7).

Additionally, our results demonstrate that the steady-state number of ERMES foci is determined by a combination of fusion, fission, *de novo* formation and inheritance events (Figs 3,4; Fig. S4). The primary mechanism of new ERMES MCS formation appears to be the *de novo* formation of new contacts. As cells grow and synthesize new tethers, small groups of tethers likely coalesce into stable contact sites. When mitochondria–PM tethering is disrupted, rather than forming new contact sites, newly synthesized tethers likely get incorporated into the enlarged contact sites. In addition, loss of cortical mitochondrial tethering will reduce the total amount of cellular space occupied by the mitochondrial network. This might increase the chance that existing ERMES contacts will become close enough to fuse into larger contacts. In sum, we hypothesize that a key role of mitochondria–PM tethering is to maintain the spatial segregation of MCSs by controlling mitochondrial distribution and morphology. Interestingly, a recent study has reported that loss of the mitochondrial division machinery, which generates a hyperfused mitochondrial network, also decreases the number but increases the size of ERMES contacts (Kakimoto-Takeda et al., 2022). Thus, there could be several mechanisms that regulate the spatial distribution of ERMES by controlling mitochondrial morphology. An attractive model is that maintaining the spatial distribution of ERMES foci throughout the cell contributes to the efficient transport of lipids between mitochondria and the ER. Rather than limiting transport to a single location and relying on diffusion to move lipids throughout the organelle network, cells can utilize several spatially distant MCSs to locally direct lipid transport where it is most needed.

Although we have primarily investigated ERMES in this study, the spatial distribution of other mitochondrial MCSs are likely regulated by mitochondria–PM tethering. Additionally, whereas the defects associated with the loss of mitochondria–PM tethering are particularly visible in budding yeast due to the dramatic morphological rearrangement of the mitochondrial network, it is likely that similar principles will apply in other organisms. Although the molecules mediating mitochondria–PM tethering in higher eukaryotes are unknown, these MCSs have been visualized in several cell types, including cardiomyocytes and retinal cells (Adams et al., 2023; Fridolfsson et al., 2012; Neto et al., 2024). We speculate that mitochondria–PM tethering might play a more important role in regulating mitochondrial function than previously appreciated.

## MATERIALS AND METHODS

### Strains and plasmids

Tables S1 and S2 list all yeast strains and plasmids used in this study. The wild-type background strain was W303 (*ade2-1; leu2-3; his3-11, 15; trp1-1; ura3-1; can1-100*; Ralser et al., 2012). All new yeast strains were generated via transformation or mating, followed by sporulation and tetrad analysis. All new plasmids were generated via standard molecular biology techniques and relevant portions were confirmed via sequencing. The plasmids used for gene deletions and tagging have been previously described (Janke et al., 2004; Longtine et al., 1998; Sheff and Thorn, 2004). All strains and constructs used in this study are available upon request.

### Imaging

For imaging, cultures were grown to mid-log phase [optical density at 600 nm (OD_600_) of 0.5–1.0] in synthetic complete plus 2% (w/v) dextrose (SCD) medium (yeast nitrogen base, US Biological; CSM powder, Sunrise Science Products) with 2× adenine (Thermo Fisher Scientific) at pH 6.4 or synthetic complete plus 3% ethanol (v/v) and 3% glycerol (v/v) (SCEG) medium (yeast nitrogen base, US Biological; CSM powder, Sunrise Science Products) with 2× adenine at pH 6.4. Cells were concentrated by centrifugation (3000 ***g*** for 1 min) and imaged on a 4% (w/v) agarose pad on a depression slide. For the 4D confocal data present in Figs 2–5 and Fig. S3, cells were adhered to concanavalin A-treated confocal dishes. Dishes were prepared as previously described (Casler and Glick, 2018). Briefly, 250 µl of 2 mg/ml concanavalin A in water was added directly to the coverslip of a confocal dish for 10 min. Dishes were washed with water and allowed to dry completely. Cells were adhered by pipetting 250 µl of a mid-log phase cell culture onto the coverslip and incubating for 15 min. Cells were gently washed with medium to remove non-adhered cells and the dish was filled with 2–3 ml of medium.

Most imaging was conducted on a Nikon SoRa spinning disk confocal system equipped with a 60×1.42 NA oil immersion objective and a Hamamatsu ORCA fusion digital CMOS camera. Images were captured with a 0.2–0.3 µm step size and deconvolved using NIS Elements (Nikon) software. All images displayed are deconvolved and whether images are projections or single slices is indicated in the figure legends. For super resolution images shown in Figs 1, 6 and Fig. S2, the same Nikon SoRa system was used with the SoRa disk in place, which enables optical photon reassignment. Super resolution images were also deconvolved using the NIS-Elements software. Additional imaging was performed on a Leica SP8 confocal equipped with HyD detectors and a 63×1.4 NA oil immersion objective. No images from this system are displayed in figures, but they were used for the quantification of ERMES foci size and intensity in Fig. 1.

### HaloTag labeling

HaloTag-tagged fusion proteins were labeled and imaged as previously described (Casler et al., 2024). JFX650 HaloTag ligand (Grimm et al., 2021) was added from a 1 mM stock in DMSO to a log-phase yeast culture in SCD pH 6.4 medium to a final concentration of 1 μM, and labeling was performed for 30 min at 24°C with shaking. Cells were washed by pushing 6 ml of fresh medium through a 0.22 μm syringe filter to remove excess dye. Washed cells were resuspended in SCD pH 6.4 medium by pipetting and imaged. All HaloTag fusion proteins in this work were conjugated to JFX650 HaloTag ligand for visualization.

### Quantification of fluorescence microscopy data

Fluorescence microscopy data was quantified as described below. All images were quantified using Fiji software (Schindelin et al., 2012).

### Number, intensity and area of Mdm12 foci

To quantify the number, intensity and area of Mdm12 foci, individual *Z*-stacks were first average projected and a background fluorescence value, obtained from regions of the image that did not contain cells, was subtracted. The Mdm12 channel was isolated, the fluorescence signal was smoothed using a Gaussian blur filter, and individual foci were segmented using the ‘Analyze Particles’ tool in Fiji software. The segmented regions of interest (ROIs) were then used to quantify the fluorescence signal and area of individual foci from a copy of the image that did not have the Gaussian blur applied. To be included in the quantification, foci needed to be larger than 4 pixels and above the background fluorescence value. This method was used to quantify the data presented in Fig. 1A–D and Fig. S1E,F. Each data set includes at least 117 cells over three imaging replicates per condition for a total of between 337 and 1835 foci analyzed. The number of Mdm12 foci per cell for Figs 5C and 6C were manually scored from maximum projected *Z*-stacks, and the researcher was aware of the experimental conditions. Each replicate contains 20 cells for a total of 60 cells over three replicates.

### ERMES dynamics

To quantify the number of *de novo* formation, fusion or fission events for the 4D confocal movies of Mdm12 and Mdm34 shown in Figs 3, 4 and Fig. S3, Z-stacks were maximum projected and manually inspected for the observed dynamic behaviors. The researcher was aware of the experimental conditions. To be counted as a *de novo* formation event, a new Mdm12 or Mdm34 focus had to form and persist above background fluorescence for at least 1 min. Putative fusion and fission events were first identified from maximum projections and then manually examined at individual slices within the *Z*-volume to ensure that only events occurring in the same *Z*-plane were counted. To be scored as a fusion event, two distinct Mdm12 or Mdm34 foci had to be visible as separate structures for at least 1 min, fuse into a larger structure and remain associated for at least one minute. To be scored as a fission event, one distinct Mdm12 or Mdm34 focus had to be visible as an individual structure for at least 1 min, divide into two smaller structures and remain separate for at least one minute.

### Mitochondria–ER overlap

For the quantification of the amount of mitochondrial signal masked by ER signal presented in Fig. 6F, the ER signal was smoothed with a Gaussian blur filter and used to create a mask. Then, the mitochondrial signal within the mask was subtracted. Quantification was performed on full *Z*-stacks.

### Western blotting

For Fig. 1D, cells were grown to mid-log phase in YPD, harvested by centrifugation (3000 ***g*** for 1 min) and whole-cell extracts were prepared by NaOH lysis and trichloroacetic acid precipitation. Pellets were resuspended in 50 µl MURB (100 mM MES, pH 7, 1% SDS and 3 M urea) and analyzed by SDS-PAGE using a 10% Bis-tris gel (Invitrogen, NP0302BOX). After transfer, blots were analyzed with Revert Total Protein Stain (LI-COR Biosciences) according to the manufacturer's protocol. Blots were then washed and probed with an anti-GFP (Abclonal, AE011) antibody at a 1:2000 dilution. A secondary goat anti-rabbit-IgG antibody (Azure Biosystems, AC2128) was used at a 1:15,000 dilution for detection. Blots were imaged on the Odyssey Infrared Imaging System (LI-COR Biosciences) and quantified using ImageStudio (LI-COR Biosciences). Molecular mass standards are PageRuler Plus Prestained Protein Ladder (Thermo Fisher Scientific 26619). The full image of the western blot in Fig. 1D is shown in Fig. S4.

### Growth assays

Serial dilution growth assays (shown in Fig. S1A) were performed by growing cells in YPD medium overnight at 30°C and pelleting 0.2 OD_600_ units in a microcentrifuge (3000 ***g*** for 1 min). Tenfold serial dilutions were spotted onto plates of the indicated medium and grown at 30°C for 2 days prior to imaging. YPD medium consists of 1% yeast extract, 2% peptone and 2% dextrose. YPEG medium consists of 1% yeast extract, 2% peptone, 3% ethanol and 3% glycerol.

## Supplementary Material



10.1242/joces.263685_sup1 Supplementary information
