# Supplementary material for: Mitochondria–plasma membrane contact sites regulate the ER–mitochondria encounter structure
Source: J Cell Sci. 2025 Feb 18;138(9):JCS263685. doi: 10.1242/jcs.263685 (PMC11883241; doi:10.1242/jcs.263685)
Supplement: Supplementary information [file joces-138-263685-s1.pdf]

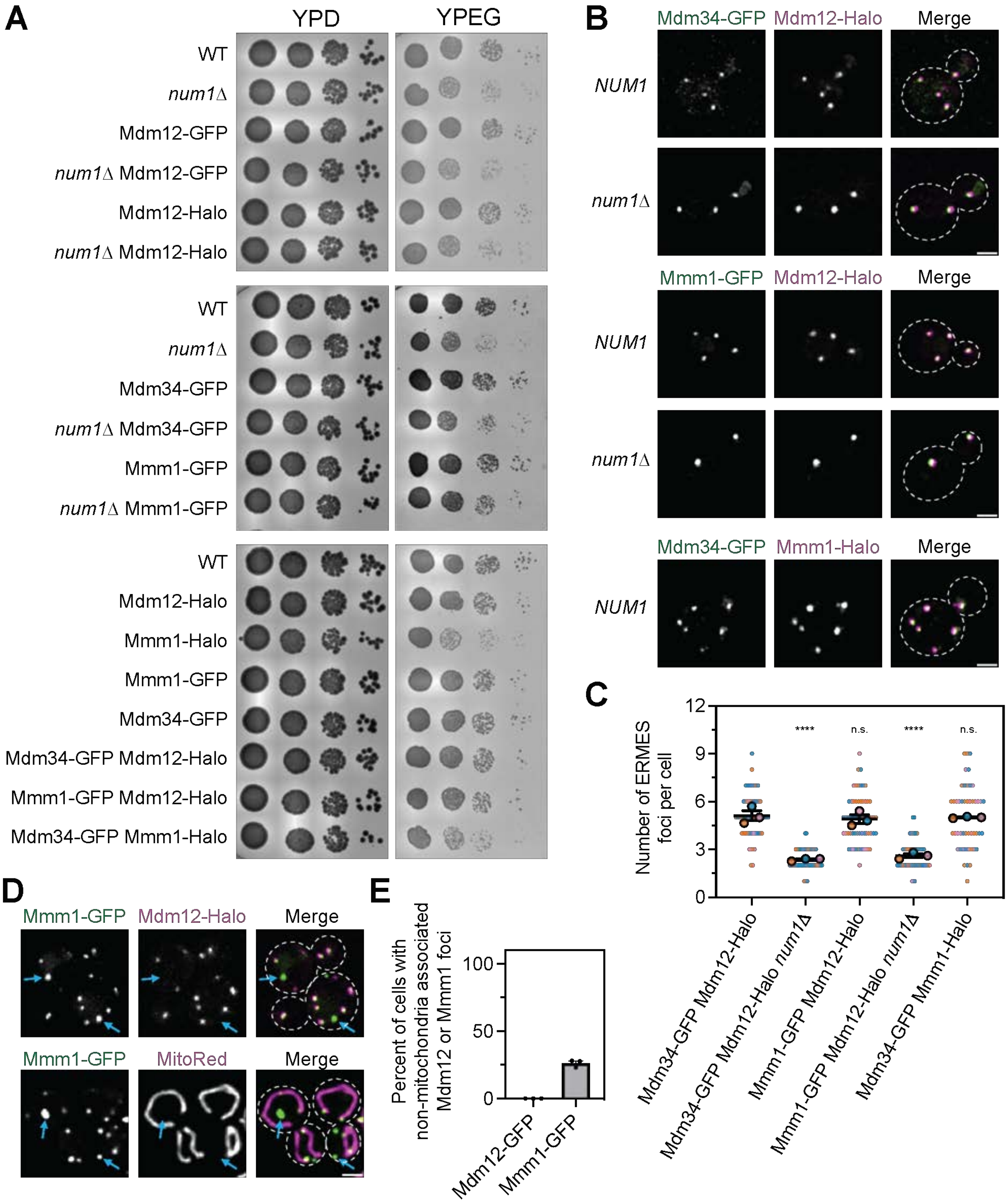

Fig. S1. Verification of the functionality of ERMES fusion proteins. (A) 10-fold serial dilutions of the indicated strains were spotted on YPD or YPEG and grown at 30°C for two days. The image is a representative example of three biological replicates. **(B)** Fluorescence micrographs of cells expressing the indicated ERMES subunit fusion proteins in *NUM1* and

Journal of Cell Science • Supplementary information

*num1*Δ backgrounds. Individual channels are shown in grayscale. Cell outlines are indicated by dashed white lines. Scale bar 2 μm. Images are max projections of full Z-stacks. **(C)** Quantification of the number of ERMES foci containing both indicated ERMES subunits from the data in (B). Quantification is depicted identically to Fig. 1B. Statistical significance was determined by an ordinary one-way ANOVA with multiple comparisons (**** = $p < 0.0001$, n.s. not significant). Statistical comparisons are in reference to the number of Mdm12 foci in wild-type cells (Fig. 1B). **(D)** Fluorescence micrographs of cells expressing Mmm1-GFP with Mdm12-Halo or MitoRed. Blue arrows highlight large Mmm1 foci that do not colocalize with Mdm12 or MitoRed. Cell outlines are indicated by dashed white lines. Scale bar 2 μm. Images are max projections of full Z-stacks. **(E)** Quantification of the percentage of cells with non-mitochondria associated Mdm12 or Mmm1 foci from the cells in (D). Each dot represents the percentage of cells that had at least one non-mitochondria associated Mdm12 or Mmm1 foci in an imaging replicate containing 100 cells. Error bars represent the SEM of the three replicates.

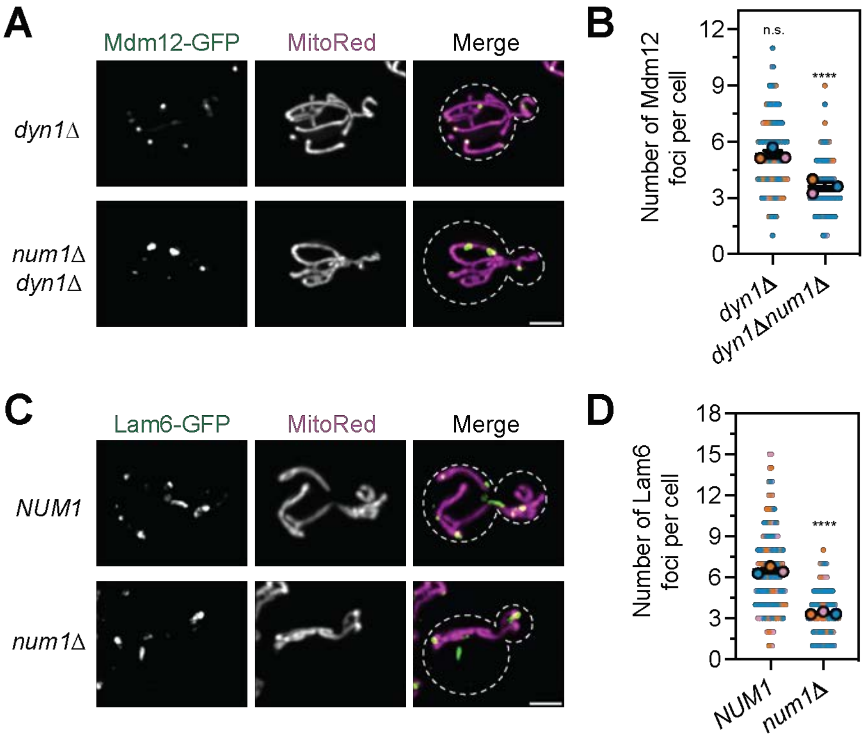

Fig. S2. The presence of dynein does not influence ERMES foci number and the number of Lam6 foci is reduced in *num1*Δ mutants. (A) Super resolution fluorescence micrographs of cells expressing Mdm12-GFP and MitoRed in *dyn1*Δ and *dyn1*Δ*num1*Δ backgrounds. Individual channels are shown in grayscale. Cell outlines are indicated by dashed lines. Scale bar 2 μm. **(B)** Quantification of the number of Mdm12 foci from the cells in (A). Quantification is depicted identically to Fig. 1 B. Statistical significance was determined by an ordinary one-way ANOVA with multiple comparisons (**** = $p < 0.0001$, n.s. not significant). Statistical significance is in comparison to the number of Mdm12 foci in wild-type cells (Fig. 1 B). **(C)** Identical to (A) except cells expressed Lam6-GFP in *NUM1* or *num1*Δ backgrounds. **(D)** Quantification of the number of Lam6 foci in (C). Quantification is depicted identically to Fig.1 B. Statistical significance was determined via an unpaired *t* test (**** = $p < 0.0001$).

Journal of Cell Science • Supplementary information

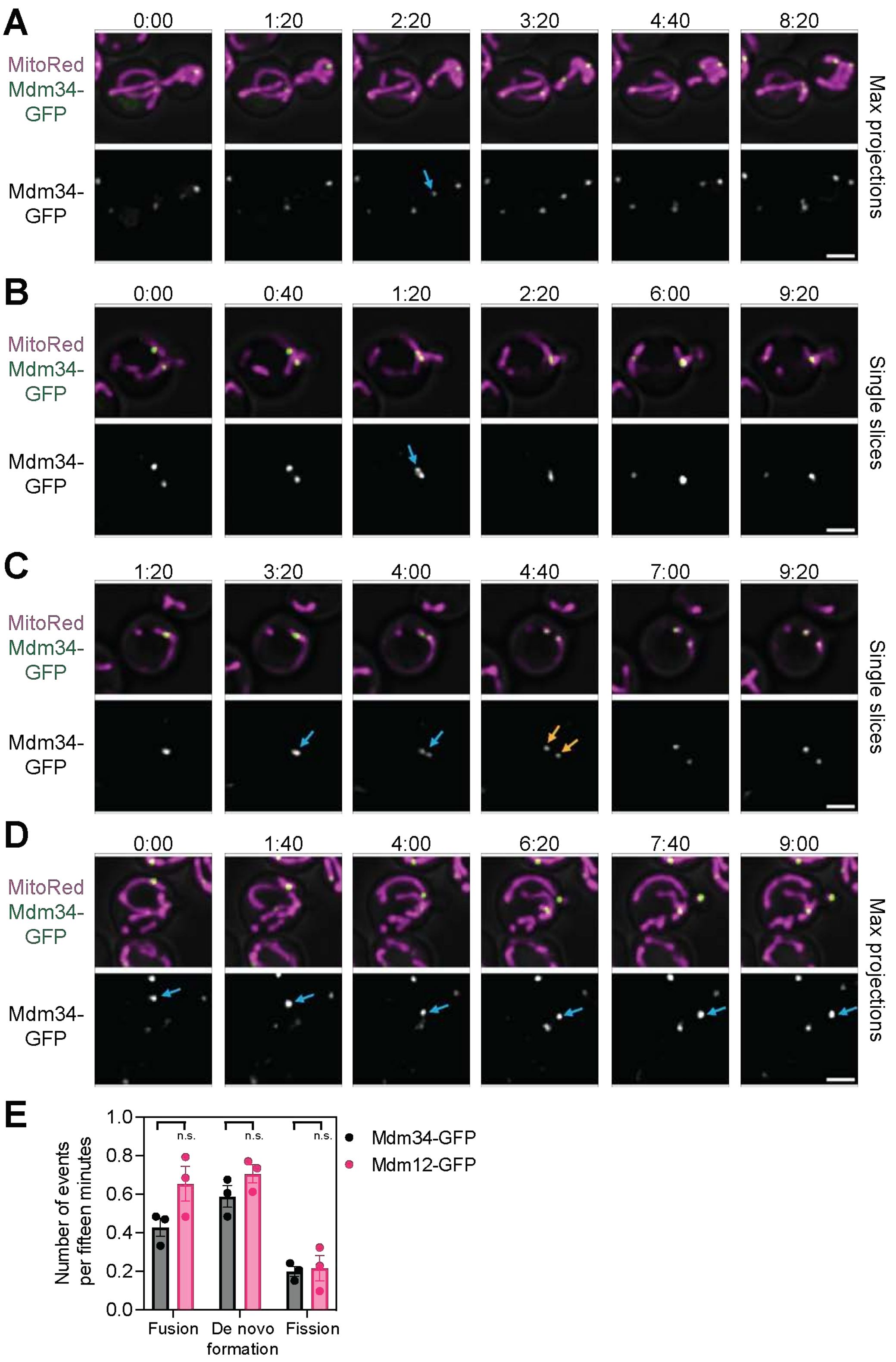

Fig. S3. Visualization of ERMES foci dynamics with an additional reporter. Cells expressing Mdm34-GFP and MitoRed were grown to mid-log phase and imaged via 4D confocal microscopy. Panels A-D are fluorescence micrographs from Movies S6-9 representing various

Journal of Cell Science • Supplementary information

observed dynamic behaviors of Mdm34 foci. **(A)** The blue arrow points to the de novo formation of a new Mdm34-GFP focus that persists throughout the rest of the video. Images are max projections of a full Z-stack. Scale bar 2 μm. **(B)** An example of two distinct ERMES foci that fuse and remain associated. Images are single slices from a Z-stack. **(C)** An example of a single Mdm34 focus (blue arrow) splitting into two smaller foci (orange arrows) that remain separated. Images are single slices from a Z-stack. **(D)** Blue arrows indicate an example of an Mdm34 focus being transported from the mother to the daughter cell. Images are max projections of a full Z-stack. **(E)** Comparison of the frequency of the indicated dynamic events for cells expressing Mdm34-GFP or Mdm12-GFP. Mdm12-GFP data is duplicated from Fig. 3 E to aid visual comparison. Statistical significance was determined via an unpaired *t* test (n.s. = not significant).

Journal of Cell Science • Supplementary information

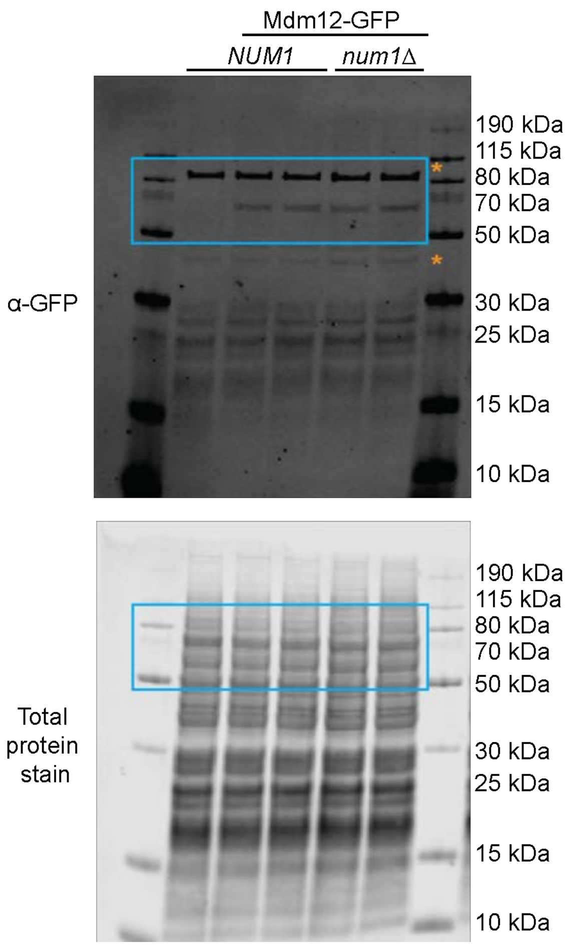

Fig. S4. Blot transparency. Full image of the western blot shown in Fig. 1D.

Table S1. Yeast strains

LLY	Genotype	Source
92	W303 (*ade2–1; leu2–3; his3–11,15; trp1–1; ura3–1; can1–100*)	
**Figure 1**		
4255	W303 *MDM12-yEGFP::HIS pRS305-mito-DsRed::LEU*	This study
4516	W303 *MDM12-yEGFP::HIS pRS305-mito-DsRed::LEU num1Δ:KAN*	This study
4908	W303 *NUM1ΔFFAT-yoHalo::URA MDM12-yEGFP::HIS pRS305-mito-DsRed::LEU*	This study
5002	W303 *MDM12-yEGFP::HIS mdm36Δ::HIS pRS305-mito-DsRed::LEU*	This study
5233	W303 *MDM12-yEGFP::HIS vps39Δ::NAT pRS305-mito-DsRed::LEU*	This study
5234	W303 *MDM12-yEGFP::HIS vps39Δ::NAT num1Δ::KAN pRS305-mito-DsRed::LEU*	This study
**Figure 2**		
4443	W303 *NUM1-yoHalo::URA MDM12-yEGFP::HIS pRS305-mito-DsRed::LEU*	This study
**Figure 3**		
5301	W303 *MDM12-yoHalo::TRP pRS305-mito-DsRed-GFP-HDEL::LEU*	This study
**Figure 5**		
5249	W303 *tor1-1 fpr1Δ::NAT MDM12-yoHalo::TRP pRS305-mito-DsRed::LEU*	This study
5252	W303 *tor1-1 fpr1Δ::NAT MDM12-yoHalo::TRP num1Δ::KAN pRS305-mito-DsRed::LEU*	This study
5253	W303 *tor1-1 fpr1Δ::NAT MDM12-yoHalo::TRP NUM1ΔPH-FRB-GFP::HIS TEF-FKBP12-NUM1PH::URA pRS305-mito-DsRed::LEU*	This study
**Figure 6**		
5258	W303 *num1Δ::HIS pRS304-CYC1-yEGFP-Mdv1(1-241)::TRP1 PIL1-LaG16::URA MDM12-yoHalo::TRP pRS305-mito-DsRed::LEU*	This study
6553	W303 *num1Δ::HIS pRS304-CYC1-yEGFP-Mdv1(1-241)::TRP1 MDM12-yoHalo::TRP pRS305-mito-DsRed::LEU*	This study
5247	W303 *num1Δ:::KAN pRS304-CYC1-Tom70mito-yEGFP::TRP1 SEG1-LaG16::URA MDM12-yoHalo::TRP pRS305-mito-DsRed::LEU*	This study
6552	W303 *num1Δ:::KAN pRS304-CYC1-Tom70mito-yEGFP::TRP1 MDM12-yoHalo::TRP pRS305-mito-DsRed::LEU*	This study
5126	W303 *MDM12-GFP::HIS pRS305-mito-DsRed::LEU TEF::MDM36::NAT*	This study
113	W303 *YIplac204-GFP-HDEL::TRP mito-DsRed::URA*	This study
115	W303 *YIplac204-GFP-HDEL::TRP mito-DsRed::URA num1Δ::HIS*	This study

	**Figure S1**	
28	W303 *num1Δ::HIS*	Lackner et al., 2013
4252	W303 *MDM12-yEGFP::HIS*	This study
4515	W303 *MDM12-yEGFP::HIS num1Δ:KAN*	This study
4337	W303 *MDM12-yoHalo::TRP*	This study
4898	W303 *MDM12-yoHalo::TRP num1Δ::KAN*	This study
5711	W303 *MDM34-yEGFP::HIS*	This study
5742	W303 *MDM34-yEGFP::HIS pRS305-mito-DsRed::LEU num1Δ::KAN*	This study
5713	W303 *MMM1-yEGFP::HIS*	This study
5747	W303 *MMM1-yEGFP::HIS pRS305-mito-DsRed::LEU num1Δ::KAN*	This study
5714	W303 *MMM1-yoHalo::URA*	This study
5741	W303 *MDM34-yEGFP::HIS MDM12-yoHalo::TRP*	This study
5745	W303 *MMM1-yEGFP::HIS MDM12-yoHalo::TRP*	This study
5749	W303 *MMM1-yoHalo::URA MDM34-yEGFP::HIS*	This study
5746	W303 *MMM1-yEGFP::HIS pRS305-mito-DsRed::LEU*	This study
**Figure S2**		
5412	W303 *MDM12-yEGFP::HIS pRS305-mito-DsRed::LEU dyn1Δ::NAT*	This study
5413	W303 *MDM12-yEGFP::HIS pRS305-mito-DsRed::LEU dyn1Δ::NAT num1Δ::HIS*	This study
5245	W303 *LAM6-yEGFP::HIS pRS305-mito-DsRed::LEU*	This study
5246	W303 *LAM6-yEGFP::HIS pRS305-mito-DsRed::LEU num1Δ::KAN pRS305-mito-DsRed::LEU*	This study
**Figure S3**		
5740	W303 *MDM34-yEGFP::HIS pRS305-mito-DsRed::LEU*	This study

Journal of Cell Science • Supplementary information

Table S2. Plasmids

LLEC	Plasmid Name	Source
19	pRS305-mito-DsRed	Abrisch et al., 2020
23	pRS305-GFP-HDEL	Casler et al., 2024
54	pKT128 pFA6a–link–yEGFP–SpHIS5	Sheff & Thorn, 2004
35	pFA6a-3HA-His3MX6	Bähler et al., 1998
743	pFA6a-yoHalo::CaUra3	Subramanian et al., 2019
27	pFA6a-kanMX6	Bähler et al., 1998
850	pFA6a-yoHalo::TRP1	Casler et al., 2024
473	pFA6a-NAT	Longtine et al., 1998
28	pFA6a-TRP1	Bähler et al., 1998
18	pRS305-mito-DsRed-GFP-HDEL	Abrisch et al., 2020
85	*YIplac204-GFP-HDEL::TRP*	Rossanese et al., 2001
84	*pVT100-mito-DsRed*::*URA*	This study

Journal of Cell Science • Supplementary information

Supplement References

Abrisch, R. G., Gumbin, S. C., Wisniewski, B. T., Lackner, L. L., & Voeltz, G. K. (2020). Fission and fusion machineries converge at ER contact sites to regulate mitochondrial morphology. *J. Cell Biol.*, *219*(4). https://doi.org/10.1083/jcb.201911122

Bähler, J., Wu, J. Q., Longtine, M. S., Shah, N. G., McKenzie, A., Steever, A. B., Wach, A., Philippsen, P., & Pringle, J. R. (1998). Heterologous modules for efficient and versatile PCR-based gene targeting in Schizosaccharomyces pombe. *Yeast*, *14*(10), 943–951. https://doi.org/10.1002/(SICI)1097-0061(199807)14:10<943::AID-YEA292>3.0.CO;2-Y

Longtine, M. S., McKenzie, A., Demarini, D. J., Shah, N. G., Wach, A., Brachat, A., Philippsen, P., & Pringle, J. R. (1998). Additional modules for versatile and economical PCR-based gene deletion and modification in Saccharomyces cerevisiae. *Yeast*, *14*(10), 953–961. https://doi.org/10.1002/(SICI)1097-0061(199807)14:10<953::AID-YEA293>3.0.CO;2-U

Rossanese, O. W., Reinke, C. a., Bevis, B. J., Hammond, A. T., Sears, I. B., O'Connor, J., & Glick, B. S. (2001). A role for actin, Cdc1p, and Myo2p in the inheritance of late Golgi elements in Saccharomyces cerevisiae. *J. Cell Biol.*, *153*(1), 47–62. https://doi.org/10.1083/jcb.153.1.47

Sheff, M. A., & Thorn, K. S. (2004). Optimized cassettes for fluorescent protein tagging in Saccharomyces cerevisiae. *Yeast*, *21*(8), 661–670. https://doi.org/10.1002/yea.1130

Subramanian, K., Jochem, A., Vasseur, M. Le, Lewis, S., Paulson, B. R., Reddy, T. R., Russell, J. D., Coon, J. J., Pagliarini, D. J., & Nunnari, J. (2019). Coenzyme Q biosynthetic proteins assemble in a substrate-dependent manner into domains at ER-mitochondria contacts. *J. Cell Biol.*, *218*(4), 1352–1368. https://doi.org/10.1083/jcb.201808044v

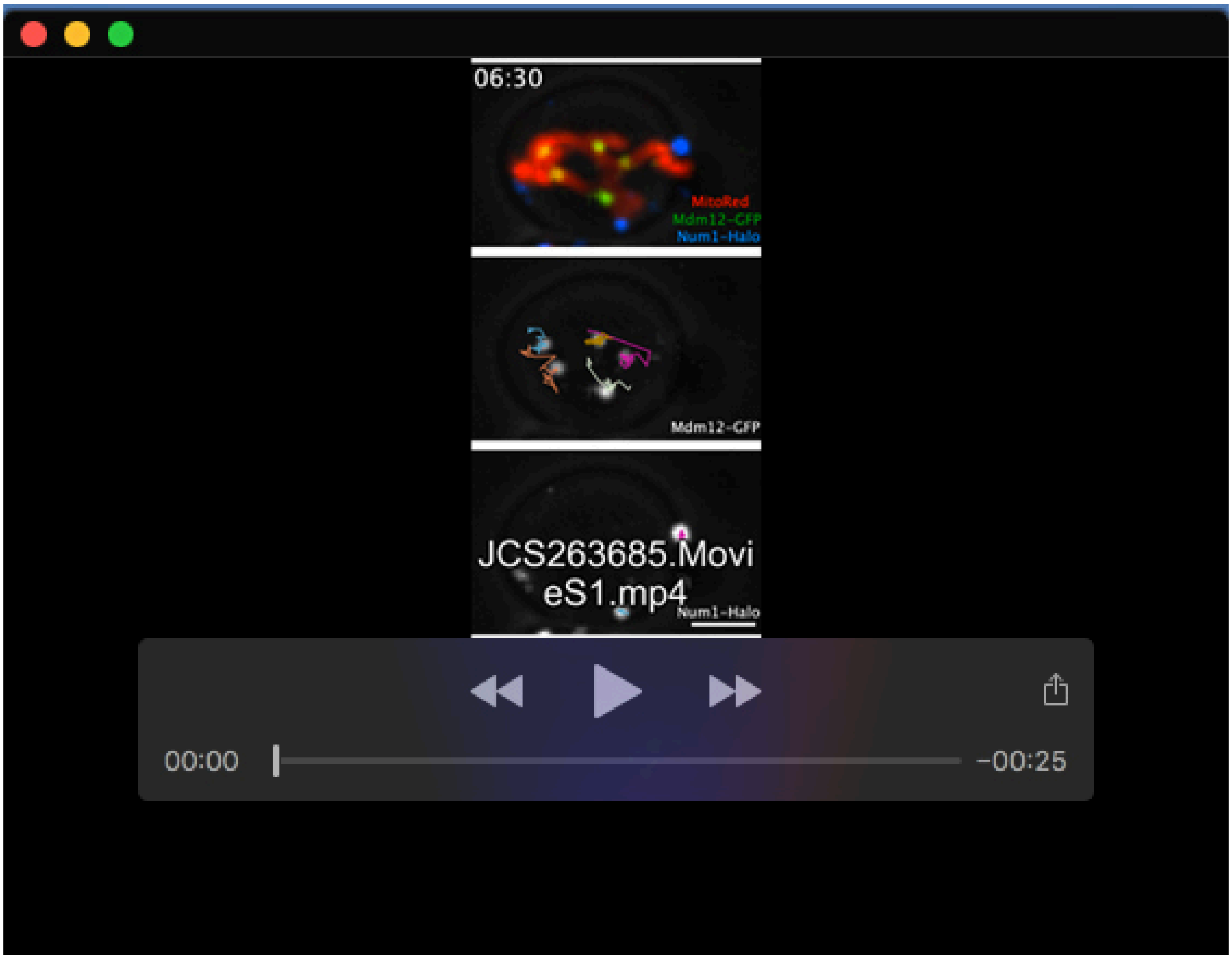

Movie 1. ERMES foci move with the mitochondrial network. Cells expressing the mitochondrial matrix marker MitoRed, Mdm12-GFP, and Num1-Halo were grown to mid-log phase, labeled with JFX650, adhered to a ConA treated confocal dish, and imaged. The movie is a max projection of a full Z-stack. Merged fluorescence channels are shown on top and the individual fluorescence channels from the Mdm12-GFP and Num1-Halo channels are shown in gray scale below. All channels are overlayed with a bright field image. The trajectories of the Num1 and Mdm12 foci were identified using the TrackMate plugin for Fiji and are indicated by a colored line overlayed on the grayscale images. Stills from this movie are shown in Fig. 2A. Scale bar 2 µm. Time is in min:s.

Journal of Cell Science • Supplementary information

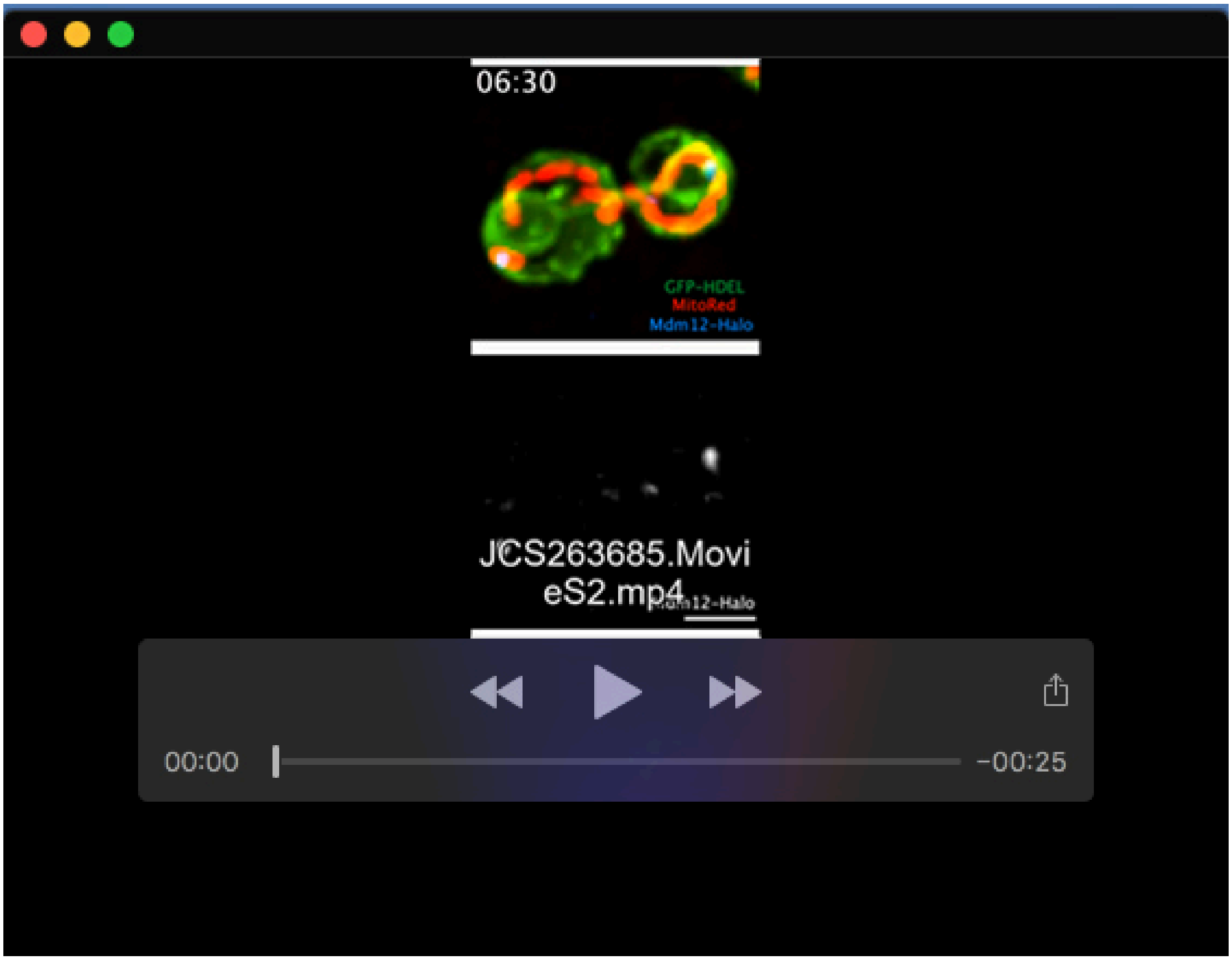

Movie 2. ERMES foci form de novo. Cells expressing Mdm12-Halo, the mitochondrial matrix marker MitoRed, and the ER marker GFP-HDEL, were grown to mid-log phase, labeled with JFX650, adhered to a ConA treated confocal dish, and imaged. The movie depicts an event where a new Mdm12 focus forms. The movie is a max projection of full Z-stacks to demonstrate that no Mdm12 focus was present in the cell at that location prior to formation. A merged image of all fluorescence channels is shown on top and the Mdm12-Halo channel is shown in grayscale below. Images from this movie are shown in Fig. 3 A. Scale bar 2 µm. Time is in min:s.

Journal of Cell Science • Supplementary information

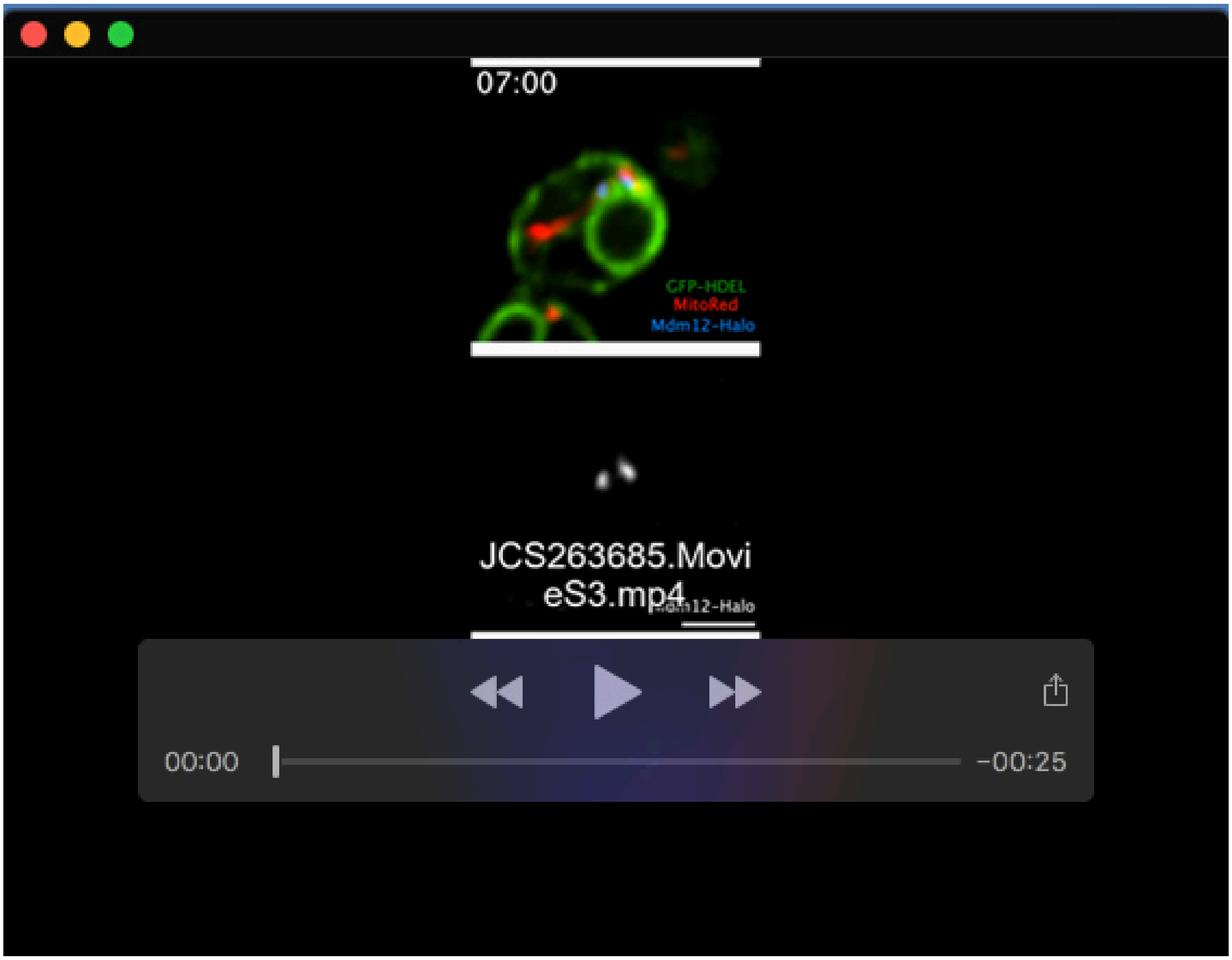

Movie 3. ERMES foci fuse. Cells were grown and imaged identically to Movie 2. The movie depicts an event where two distinct Mdm12 foci approach and fuse into a single focus. The movie is an individual slice from a full Z-stack to demonstrate the fusion event is happening on the same imaging plane. A merged image of all fluorescence channels is shown on top and the Mdm12-Halo channel is shown in grayscale below. Images from this movie are shown in Fig. 3 B. Scale bar 2 μm. Time is in min:s.

Journal of Cell Science • Supplementary information

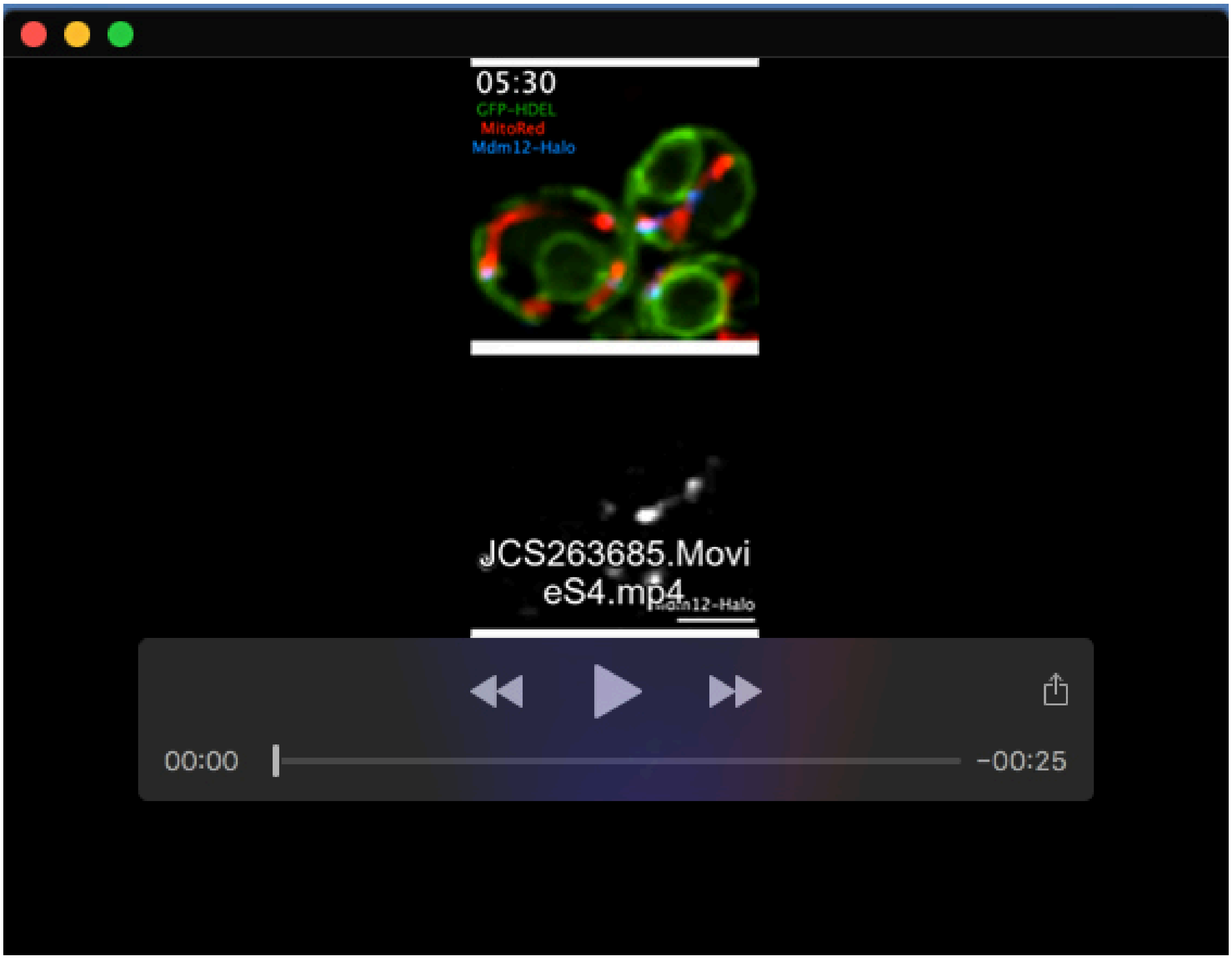

Movie 4. ERMES foci divide. Cells were grown and imaged identically to Movie 2. The movie depicts an event where one distinct Mdm12 focus divides into two distinct foci. The movie is an individual slice from a full Z-stack to demonstrate the fission event is happening on the same imaging plane. A merged image of all fluorescence channels is shown on top and the Mdm12-Halo channel is shown in grayscale below. Images from this movie are shown in Fig. 3C. Scale bar 2 µm. Time is in min:s.

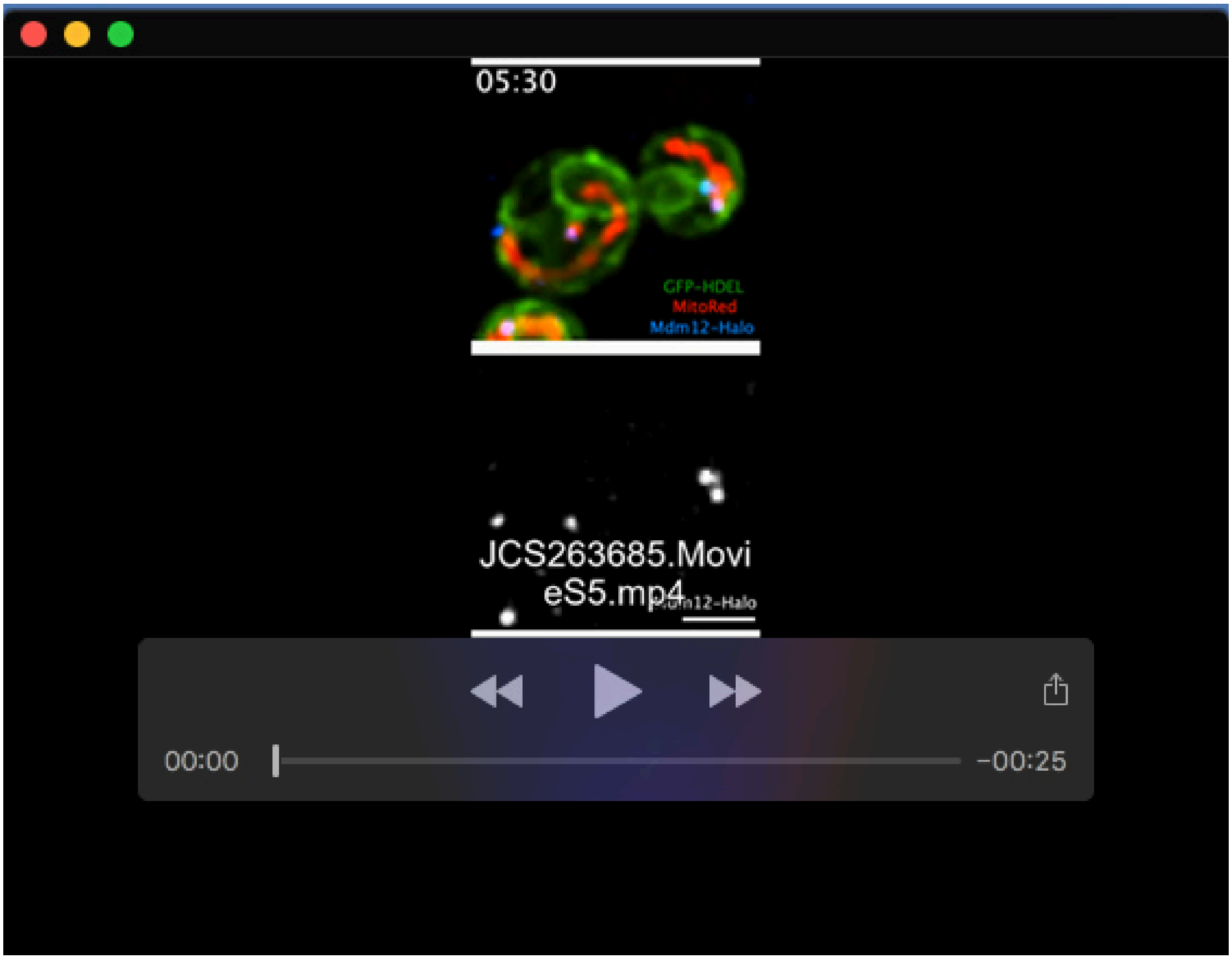

Movie 5. ERMES foci can be inherited. Cells were grown and imaged identically to Movie 2. The movie depicts an event where a Mdm12 focus moves from the mother cell to the daughter cell. The movie is a max projection of a full Z-stack. A merged image of all fluorescence channels is shown on top and the Mdm12-Halo channel is shown in grayscale below. Images from this movie are shown in Fig. 3 D. Scale bar 2 µm. Time is in min:s.

Journal of Cell Science • Supplementary information

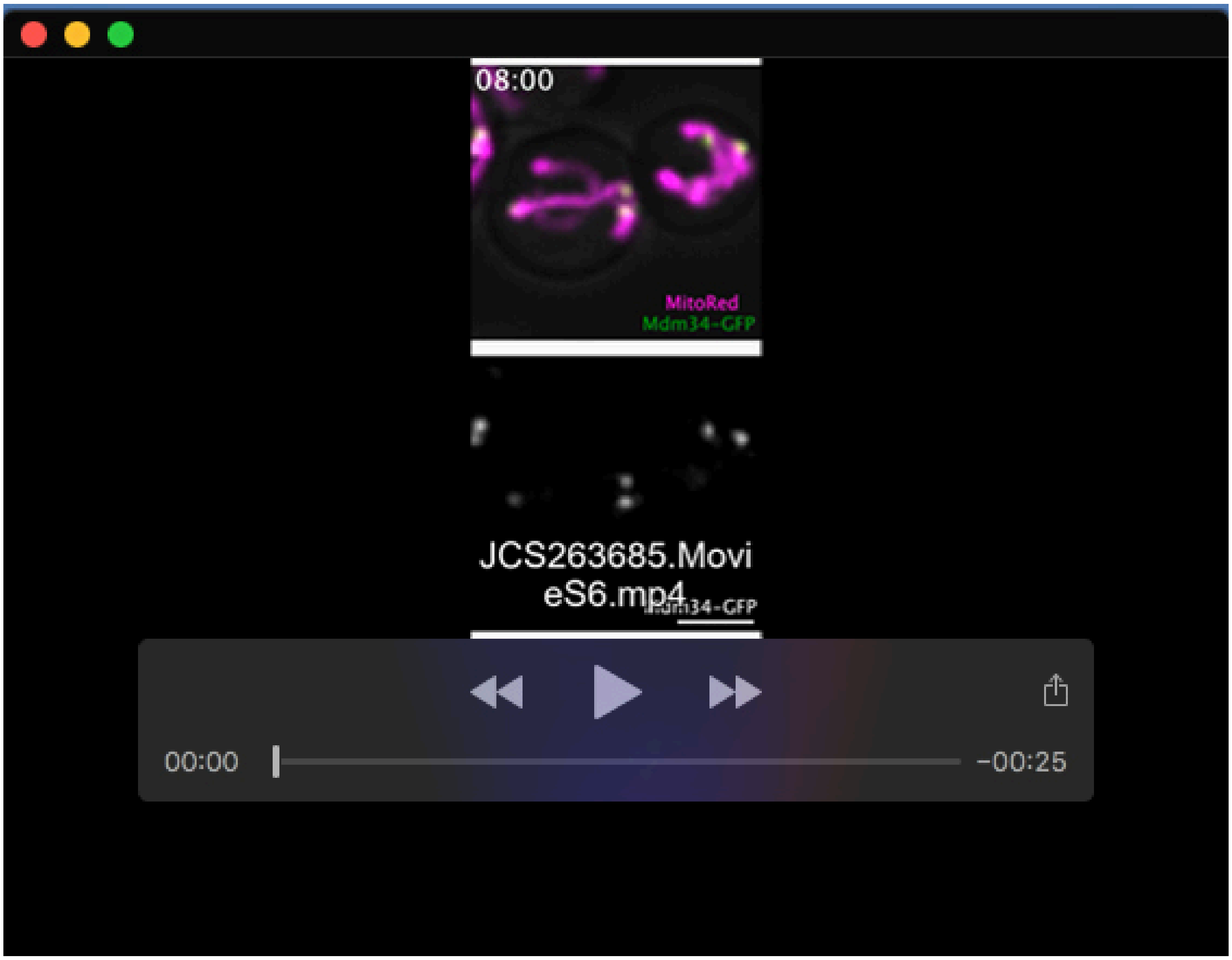

Movie 6. Mdm34 foci form de novo. Cells expressing Mdm34-GFP and MitoRed were grown to mid-log phase, adhered to ConA treated confocal dishes, and imaged. The movie depicts an event where a new Mdm34 focus forms de novo. The movie is a max projection of full Z-stacks to demonstrate that no Mdm34 focus was present in the cell at that location prior to formation. A merged image of the fluorescence channels and a bright field image is shown on top and the Mdm34-GFP channel is shown in grayscale below. Images from this movie are shown in Fig. S4 A. Scale bar 2 µm. Time is in min:s.

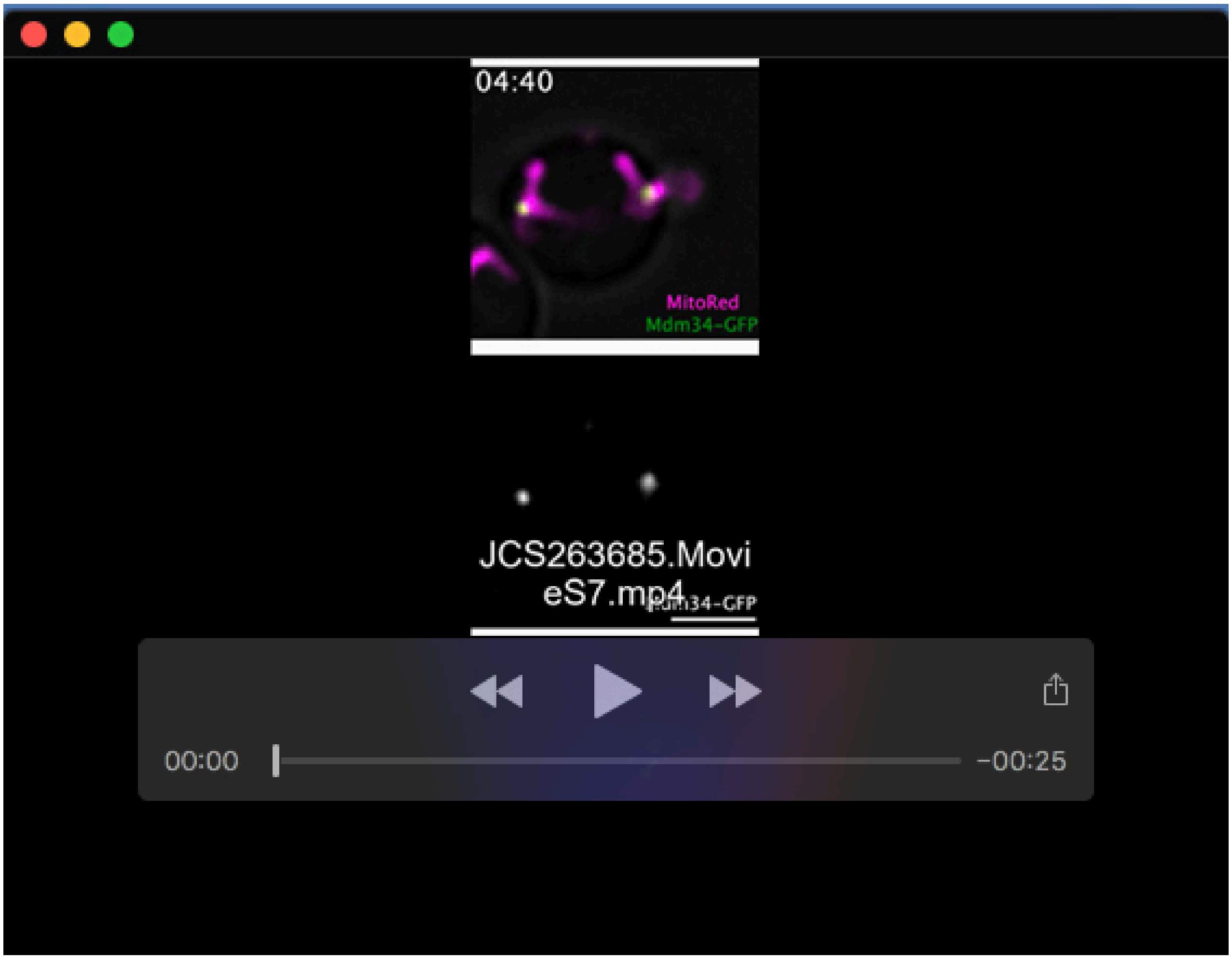

Movie 7. Mdm34 foci fuse. The movie was captured and is presented identically to Movie 6. The movie depicts an event where two Mdm34 foci fuse. The movie is an individual slice from a full Z-stack to demonstrate the fusion event is happening on the same imaging plane. Images from this movie are shown in Fig. S4B. Scale bar 2 μm. Time is in min:s.

Journal of Cell Science • Supplementary information

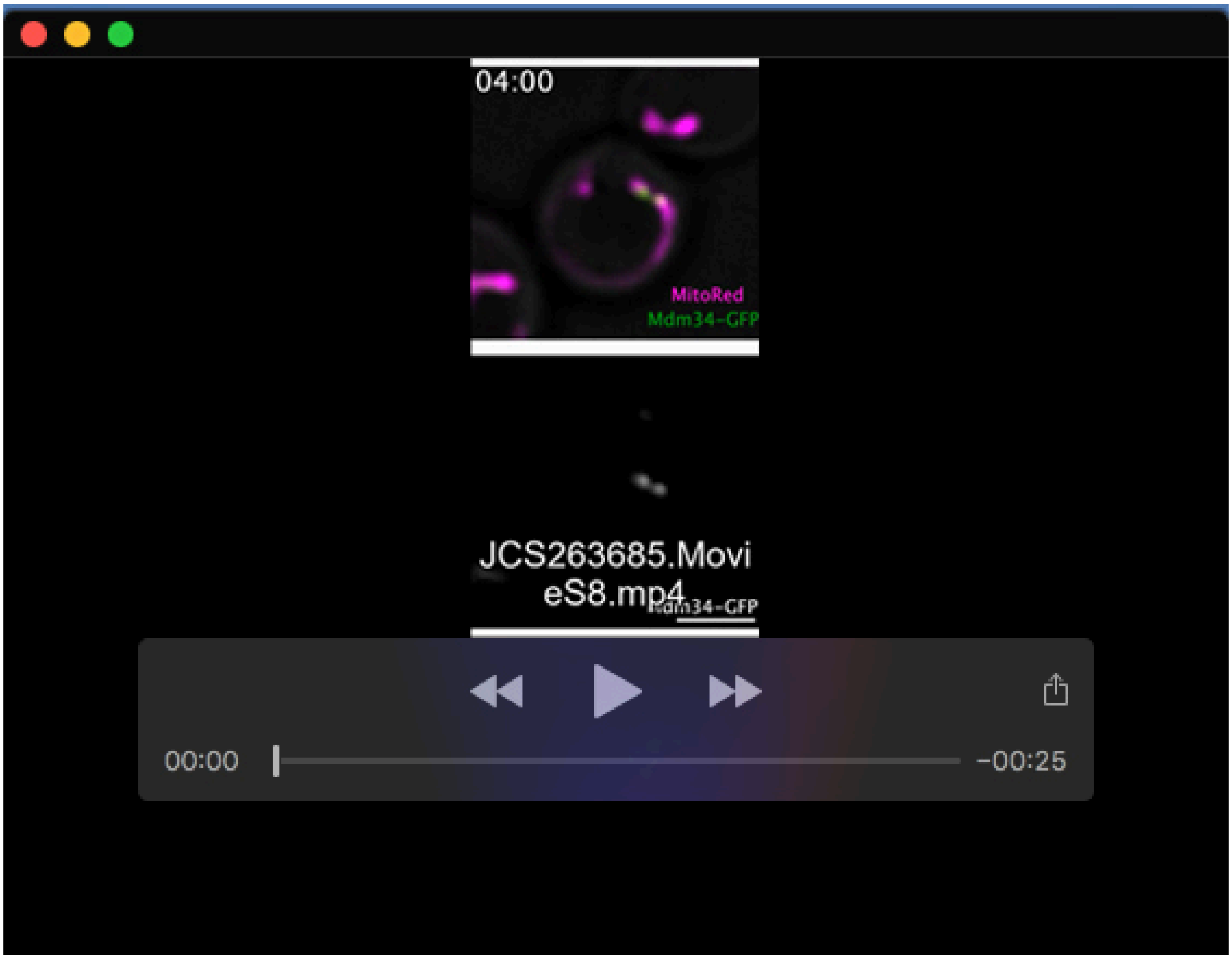

Movie 8. Mdm34 foci divide. The movie was captured and is presented identically to Movie 6. The movie depicts an event where one Mdm34 focus divides into two distinct foci. The movie is an individual slice from a full Z-stack to demonstrate the fission event is happening on the same imaging plane. Images from this movie are shown in Fig. S4C. Scale bar 2 μm. Time is in min:s.

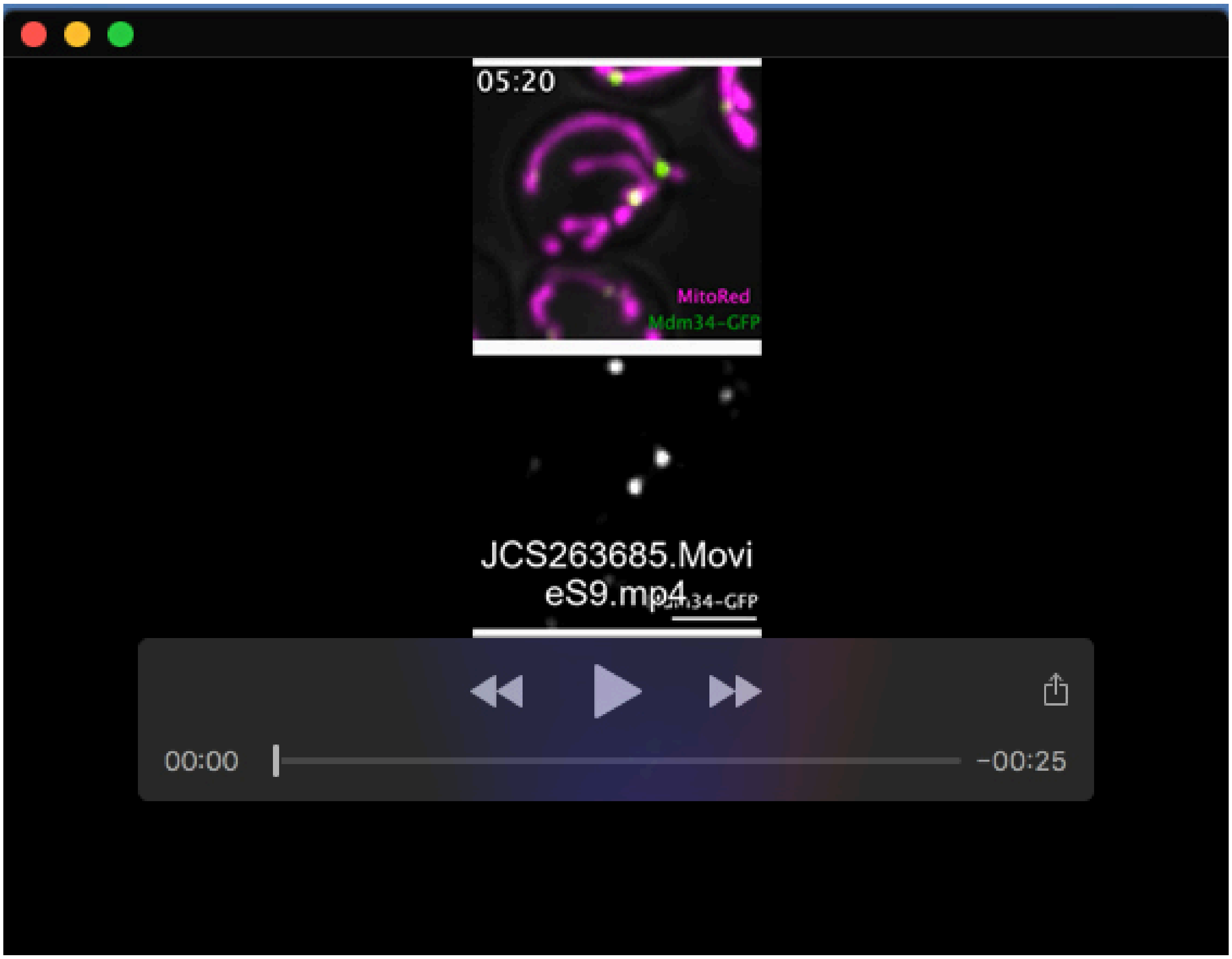

Movie 9. Mdm34 foci can be inherited. The movie was captured and is presented identically to Movie 6. The movie depicts an event where an Mdm34 focus is transported from the mother to the growing bud. The movie is a max projection of full Z-stacks. Images from this movie are shown in Fig. S4D. Scale bar 2 µm. Time is in min:s.

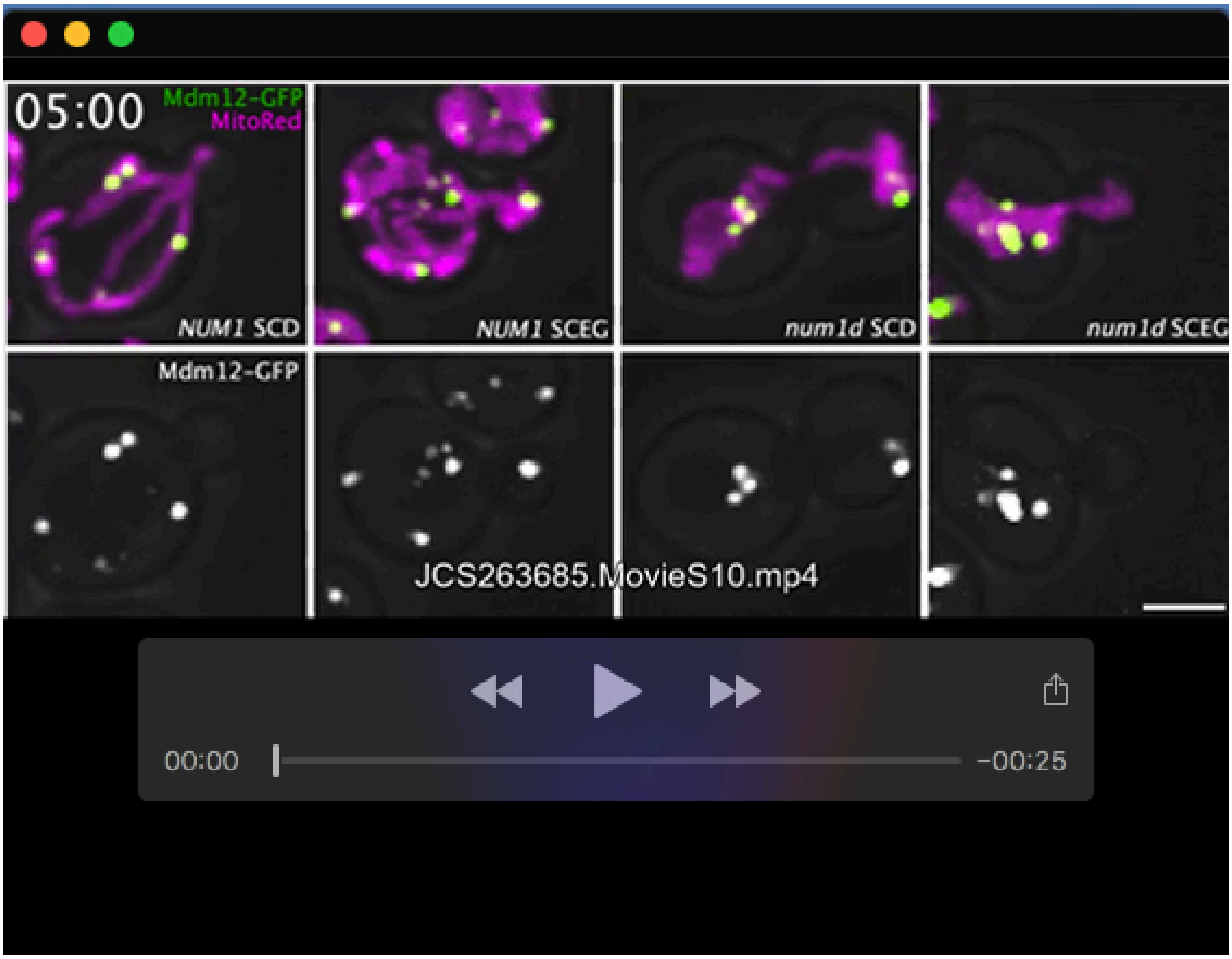

Movie 10. Loss of MECA and changes in growth conditions modulate the rates of ERMES dynamics. Cells expressing Mdm12-GFP and MitoRed in wild-type or *num1*Δ backgrounds were grown in SCD or SCEG to mid-log phase, adhered to ConA treated confocal dishes, and imaged. Representative examples of each genetic background and growth condition are tiled together to aid visual comparison. A merged fluorescence channel is shown on top and the individual Mdm12-GFP channel is shown in grayscale below. Fluorescence channels are overlayed with a bright field image. All movies are max projections of full Z-stacks. Images from this movie are shown in Fig. 4 A-D. Scale bar 2 μm. Time is in min:s.

Journal of Cell Science • Supplementary information
