# Supplementary material for: Mitochondria–plasma membrane contact sites regulate the ER–mitochondria encounter structure
Source: J Cell Sci. 2025 Feb 18;138(9):JCS263685. doi: 10.1242/jcs.263685 (PMC11883241; doi:10.1242/jcs.263685)
Supplement: Supplementary file 1 [file joces-138-263685_review_history.pdf]

Mitochondria-plasma membrane contact sites regulate the ER-mitochondria encounter structure

Jason C. Casler, Clare Harper and Laura L. Lackner
DOI: 10.1242/jcs.263685

Editor: Heidi McBride

Review timeline
Original submission: 4 November 2024
Editorial decision: 6 December 2024
First revision received: 31 December 2024
Accepted: 8 January 2025

Original submission

First decision letter

MS ID#: jcs.263685
MS TITLE: Mitochondria-plasma membrane contact sites regulate the ER-mitochondria encounter structure

Jason C. Casler, Clare Harper and Laura L. Lackner

We have now reached a decision on the above manuscript.

To see the reviewers' reports and a copy of this decision letter, please go to: https://submit-jcs.biologists.org and click on the 'Manuscripts with Decisions' queue in the Author Area. (Corresponding author only has access to reviews.)

As you will see, the reviewers raise a number of substantial criticisms that prevent me from accepting the paper at this stage. They suggest, however, that a revised version might prove acceptable, if you can address their concerns. If you think that you can deal satisfactorily with the criticisms on revision, I would be pleased to see a revised manuscript. We would then return it to the reviewers.

Reviewer 1

Advance summary and potential significance to field

Biogenesis and function of cellular organelles largely depend on their interactions with other organelles. In their present study, Casler et al. examined whether ERMES, the ER-mitochondria encounter structure, and MECA, the mitochondria-ER-cortex anchor, mutually determine their behavior in yeast cells. They find that loss of MECA results in a reduction of the number and at the same time in an increase of the size of ERMES foci. Furthermore, they report that the dynamics of ERMES in terms of fusion, fission and motility is altered and that these changes depend on the mitochondria-plasma membrane tethering function of MECA, rather than its mitochondria-ER tethering activity. The authors suggest that intracellular distribution of the mitochondrial network is an important factor that determines the formation of mitochondria-ER contacts. This is an interesting study, the experiments are technically sound and the manuscript is well written. A few points could be considered by the authors, as outlined below.

© 2025. Published by The Company of Biologists under the terms of the Creative Commons Attribution License (https://creativecommons.org/licenses/by/4.0/).

Comments for the author

1. The introduction and discussion of MECA is somewhat biased towards the authors' own work. They should mention, discuss and cite also the work of other groups that have contributed to the discovery and characterization of MECA and its components.
2. I'm not sure whether it makes sense to measure the area of ERMES foci because the size of ERMES is well below the resolution limit of the microscope. Determination of fluorescence intensity is a more appropriate measure. I suggest omitting Fig. 1D (which is anyway largely redundant with Fig. 1C).
3. Significance of changes of Mdm12 foci fluorescence intensity in mutants should be calculated also in comparison to WT in Fig. 1C.
4. The sentence "Remarkably, in cells lacking Num1, the increase in Mdm12 foci in both conditions was significantly reduced" is confusing and should be rephrased (lines 161-162).
5. Data in Figs. 3E and 4E suggest that fusion events of ERMES complexes are 2-3 fold more frequent than fission events. However, it would be expected that these events are balanced at steady state conditions. How can this be explained? Have the authors also observed disappearance of ERMES foci by degradation or other forms of turn-over?
6. The authors suggest in the discussion that a well distributed mitochondrial network will have more opportunities to form functional MCSs by increasing the chance it encounters other organelles, which then might increase the number of ERMES foci (lines 302-312). This could be tested by quantification of colocalization of MitoRed with GFP-HDEL in WT and num1-delta cells. An experiment along these lines would further strengthen the manuscript

<u>Reviewer 2</u>

Advance summary and potential significance to field

This paper demonstrates the importance of MECA, i.e., mitochondria-PM contact, as a key factor in regulating the number and size of ERMES foci localized at the ER-mitochondria contact sites. Using 4D live-cell imaging, the authors provide a detailed analysis of the de novo formation, fusion, and fission of ERMES, offering intriguing results that contribute significantly to our understanding of the regulatory mechanisms controlling ERMES foci. The experimental and analytical methods are sound, and the quality of the data is high.
However, the major concern with this paper is the lack of investigation into the potential impact of Num1 deficiency, which is known to inhibit mitochondrial fission. This issue should be addressed before the paper is accepted for publication

Comments for the author

Previous studies have reported that Num1 deficiency leads to impaired mitochondrial fission and that the mitochondrial fission deficiency results in a reduction in ERMES foci number and an increase in their size. Indeed, the data in the present study also show that in num1Δ and mdm36Δ cells, mitochondria are highly interconnected (Fig. 1). Could the reduced number of ERMES foci be attributed to increased encounters of ERMES on mitochondria that are highly fused by MECA deficiency, thereby promoting fusion of ERMES foci? In fact, it appears that the expression of either the iRID-Num1 system or Mito-PM artificial tether system restores abnormal connected mitochondrial shape observed in num1Δ cells, perhaps due to enhanced mitochondrial fission (Fig. 5B, Fig. 6A, B). These results suggest the possibility that MECA deficiency influences ERMES foci numbers through the inhibition of mitochondrial fission.
Would it be possible to design experiments that can separate the effects of MECA's Mito-PM tethering from mitochondrial fission? For example, what would happen to ERMES foci if you restore Mito-PM contact in num1Δ dnm1Δ cells, where mitochondrial fission is impaired, by expressing either the iRID-Num1 system or the Mito-PM artificial tether system? It might also be interesting to observe ERMES foci numbers when artificial Mito-PM tethering is enhanced in WT or dnm1Δ cells. Additionally, the authors could explore whether promoting mitochondrial fission through drug (CCCP?) treatment increases the number of ERMES foci in num1Δ cells.

© 2025. Published by The Company of Biologists under the terms of the Creative Commons Attribution License (https://creativecommons.org/licenses/by/4.0/).

First revision

Author response to reviewers' comments

Point-by-point response:

All changes made to the manuscript have been highlighted in yellow.

Reviewer 1:

Biogenesis and function of cellular organelles largely depend on their interactions with other organelles. In their present study, Casler et al. examined whether ERMES, the ER-mitochondria encounter structure, and MECA, the mitochondria-ER-cortex anchor, mutually determine their behavior in yeast cells. They find that loss of MECA results in a reduction of the number and at the same time in an increase of the size of ERMES foci. Furthermore, they report that the dynamics of ERMES in terms of fusion, fission and motility is altered and that these changes depend on the mitochondria-plasma membrane tethering function of MECA, rather than its mitochondria-ER tethering activity. The authors suggest that intracellular distribution of the mitochondrial network is an important factor that determines the formation of mitochondria-ER contacts. This is an interesting study, the experiments are technically sound and the manuscript is well written. A few points could be considered by the authors, as outlined below.

We thank the reviewer for their positive comments about the study and manuscript.

1. The introduction and discussion of MECA is somewhat biased towards the authors' own work. They should mention, discuss and cite also the work of other groups that have contributed to the discovery and characterization of MECA and its components.

We apologize for this oversight. The manuscript has been modified to include references to critical works that helped define the mechanisms of formation and functions of MECA.

2. I'm not sure whether it makes sense to measure the area of ERMES foci because the size of ERMES is well below the resolution limit of the microscope. Determination of fluorescence intensity is a more appropriate measure. I suggest omitting Fig. 1D (which is anyway largely redundant with Fig. 1C).

We agree with the reviewer and have omitted the original Fig. 1 D.

3. Significance of changes of Mdm12 foci fluorescence intensity in mutants should be calculated also in comparison to WT in Fig. 1C.

The requested statistical comparisons have been included in a revised version of Fig. 1 C.

4. The sentence "Remarkably, in cells lacking Num1, the increase in Mdm12 foci in both conditions was significantly reduced" is confusing and should be rephrased (lines 161-162).

We agree that the original language was confusing and the sentence has been replaced with: “Remarkably, loss of MECA reduced the number of ERMES foci by nearly half in both conditions.”

5. Data in Figs. 3E and 4E suggest that fusion events of ERMES complexes are 2-3 fold more frequent than fission events. However, it would be expected that these events are balanced at steady state conditions. How can this be explained? Have the authors also observed disappearance of ERMES foci by degradation or other forms of turn-over?

A potential biological explanation for the lack of balance between ERMES foci fusion and fission events is that, after fusion, small subsets of tethers may be lost from the enlarged foci, either by diffusion or small fission events, that are below the threshold of detection of our imaging systems. Additionally, the combined rates of fission and de novo formation compared to the rate of fusion would still lead to a net gain in the total number of ERMES foci over time. We did not detect any examples of ERMES foci disappearing completely in our movies. We have added text to

© 2025. Published by The Company of Biologists under the terms of the Creative Commons Attribution License (https://creativecommons.org/licenses/by/4.0/).

the manuscript (lines 233-237) to address these concerns.

6. The authors suggest in the discussion that a well distributed mitochondrial network will have more opportunities to form functional MCSs by increasing the chance it encounters other organelles, which then might increase the number of ERMES foci (lines 302-312). This could be tested by quantification of colocalization of MitoRed with GFP-HDEL in WT and num1-delta cells. An experiment along these lines would further strengthen the manuscript.

We appreciate the suggestion of this experiment. We have quantified the colocalization of mitochondria and ER in both wild type and *num1Δ* mutants. These results demonstrate that the total amount of mitochondria signal that overlaps the ER signal is reduced upon loss of MECA. These data have been included in Fig. 6 E and F in the revised manuscript.

Reviewer 2:

This paper demonstrates the importance of MECA, i.e., mitochondria-PM contact, as a key factor in regulating the number and size of ERMES foci localized at the ER-mitochondria contact sites. Using 4D live-cell imaging, the authors provide a detailed analysis of the de novo formation, fusion, and fission of ERMES, offering intriguing results that contribute significantly to our understanding of the regulatory mechanisms controlling ERMES foci. The experimental and analytical methods are sound, and the quality of the data is high. However, the major concern with this paper is the lack of investigation into the potential impact of Num1 deficiency, which is known to inhibit mitochondrial fission. This issue should be addressed before the paper is accepted for publication.

Previous studies have reported that Num1 deficiency leads to impaired mitochondrial fission and that the mitochondrial fission deficiency results in a reduction in ERMES foci number and an increase in their size. Indeed, the data in the present study also show that in num1Δ and mdm36Δ cells, mitochondria are highly interconnected (Fig. 1). Could the reduced number of ERMES foci be attributed to increased encounters of ERMES on mitochondria that are highly fused by MECA deficiency, thereby promoting fusion of ERMES foci? In fact, it appears that the expression of either the iRID-Num1 system or Mito-PM artificial tether system restores abnormal connected mitochondrial shape observed in num1Δ cells, perhaps due to enhanced mitochondrial fission (Fig. 5B, Fig. 6A, B). These results suggest the possibility that MECA deficiency influences ERMES foci numbers through the inhibition of mitochondrial fission.

Would it be possible to design experiments that can separate the effects of MECA's Mito-PM tethering from mitochondrial fission? For example, what would happen to ERMES foci if you restore Mito-PM contact in num1Δdnm1Δ cells, where mitochondrial fission is impaired, by expressing either the iRID-Num1 system or the Mito-PM artificial tether system? It might also be interesting to observe ERMES foci numbers when artificial Mito-PM tethering is enhanced in WT or dnm1Δ cells. Additionally, the authors could explore whether promoting mitochondrial fission through drug (CCCP?) treatment increases the number of ERMES foci in num1Δ cells.

We thank the reviewer for their positive comments and support of the conclusions of this manuscript. We also appreciate the interesting suggestions to try to disentangle the roles of Num1 in promoting mitochondrial division and mitochondria-PM tethering. We believe our data argues against Num1's role in regulating mitochondrial division as being a key factor in regulating the number of ERMES foci. Recently, we published two papers that strongly support a model where Num1's role in promoting mitochondrial division is related to its function as a mitochondria-ER tether, rather than a mitochondria-PM tether. In Harper et al., 2023 we demonstrated that expression of Num1-independent mitochondria-PM tethers are not sufficient to rescue the mitochondrial division defects observed upon loss of Num1. In a follow up manuscript, Casler et al., 2024, we identified Num1ΔFFAT as a separation of function allele that cleanly separates Num1's functions in mitochondria-ER and mitochondria-PM tethering. Despite maintaining cortically distributed mitochondria, the rate of mitochondrial division in cells expressing Num1ΔFFAT is reduced to the same extent as in *num1Δ* cells. In our new work, we demonstrate that cells expressing Num1ΔFFAT have wild-type numbers of ERMES foci - strongly suggesting that the redistribution of ERMES foci in a *NUM1* deletion is due to the loss of mitochondria-PM tethering

© 2025. Published by The Company of Biologists under the terms of the Creative Commons Attribution License (https://creativecommons.org/licenses/by/4.0/).

rather than changes in mitochondrial division rates. Since these lines of reasoning were not properly articulated in the original version of the manuscript, we have modified the text (lines 151-163) to address these issues.

Again, we thank the reviewer for significantly enhancing the clarity of our manuscript.

Second decision letter

MS ID#: jcs.263685R1

MS Title: Mitochondria-plasma membrane contact sites regulate the ER-mitochondria encounter structure

Authors: Jason C. Casler, Clare Harper and Laura L. Lackner

Thank you so much for submitting your study to our special issue. I am happy to tell you that your manuscript has been accepted for publication in Journal of Cell Science, pending standard publication integrity checks. It was accepted on 08 Jan 2025. Where referee reports on this version are available, they are appended below.

Comments from the Reviewers:

Reviewer 1: The authors have responded to my previous concerns in an adequate manner. Publication of this interesting manuscript can now be recommended.

Reviewer 2: The authors have provided clear and satisfactory explanations in response to my comments and concerns.

I recommend the manuscript for acceptance.

© 2025. Published by The Company of Biologists under the terms of the Creative Commons Attribution License (https://creativecommons.org/licenses/by/4.0/).
